# Population-level allelic dispersion modeling by *maelstRom* yields genome-wide maps of allele-specific dysregulation during early carcinogenesis

**DOI:** 10.1093/gigascience/giaf125

**Published:** 2025-11-04

**Authors:** Cedric Stroobandt, Louis Coussement, Tine Goovaerts, Femke De Graeve, Jeroen Galle, Wim Van Criekinge, Tim De Meyer

**Affiliations:** Department of Data Analysis and Mathematical Modelling, Ghent University, Ghent 9000, Belgium; Department of Data Analysis and Mathematical Modelling, Ghent University, Ghent 9000, Belgium; Department of Data Analysis and Mathematical Modelling, Ghent University, Ghent 9000, Belgium; Research group for Media, Innovation and Contemporary Technologies, Ghent University, Ghent 9000, Belgium; Department of Data Analysis and Mathematical Modelling, Ghent University, Ghent 9000, Belgium; Department of Data Analysis and Mathematical Modelling, Ghent University, Ghent 9000, Belgium; Department of Data Analysis and Mathematical Modelling, Ghent University, Ghent 9000, Belgium; Cancer Research Institute Ghent (CRIG), Ghent 9000, Belgium; Bioinformatics Institute Ghent N2N, Ghent University, Ghent 9000, Belgium; Department of Data Analysis and Mathematical Modelling, Ghent University, Ghent 9000, Belgium; Cancer Research Institute Ghent (CRIG), Ghent 9000, Belgium; Bioinformatics Institute Ghent N2N, Ghent University, Ghent 9000, Belgium

**Keywords:** beta-binomial distribution, expectation-maximization, mixture modeling, correlation-corrected *P* value aggregation, Lancaster method, Fisher method, clustered protocadherins

## Abstract

**Background:**

Since its inception, RNA sequencing has been pivotal in studying differential gene expression. Despite its extensive results in large-scale oncological studies, differential expression predominantly reflects a response to cancer. Therefore, we introduce differential allelic dispersion (AD) as a more effective measure. AD highlights consistent differences in expression between the 2 alleles of a gene that is, unlike *cis*-expression quantitative trait loci, independent of normal genetic variation. Such differences can, for example, arise from prevalent copy number alterations or epimutations occurring in the original cancer cell, which are mitotically expanded during cancer growth, making increased AD a marker for allele-specific dysregulation in early carcinogenesis.

**Findings:**

We present the *maelstRom* R/C++ software package that enables (differential) AD analysis solely requiring large-scale RNA sequencing data. Using the The Cancer Genome Atlas renal clear cell carcinoma cohort as a case study, we successfully benchmark *maelstRom*’s AD modeling using known copy number alterations. We also detect increased AD for loci featuring normal random monoallelic expression, including the X chromosome, but demonstrate minimal interference with cancer-specific AD detection. Finally, we identify early dysregulated genes (e.g., FBP1, CCDC8, ECHS1, CLDN7) and pathways in renal cancer, often related to metabolism (e.g., pentose phosphate pathway). Strikingly, many of these genes are known causal contributors to renal carcinogenesis.

**Conclusions:**

Differential AD clearly indicates early dysregulation in renal cancer, complementing basic differential expression analysis in cancer transcriptomics. AD is also relevant to study random monoallelic expression and may equally detect allele-specific (dys)regulation during early development or in noncancer diseases. *maelstRom* is available as an open-source software package at github.com/Biobix/maelstRom.

## Graphical Abstract

**Graphical Abstract:**
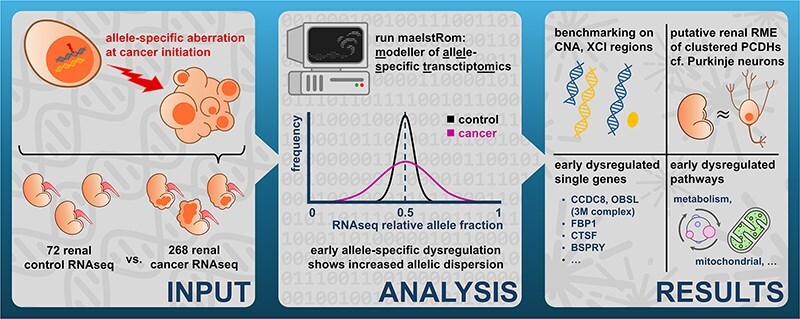

## Findings

### Background

Since its inception in the mid-2000s, RNA sequencing (RNA-seq) has primarily been used to study differential gene expression [1–3]. The continuous decrease in sequencing costs now enables population-scale biomedical studies of the entire transcriptome. Notably, large cancer cohort studies often report differential expression (DE) for over 50% of analyzed genes [4]. However, these results predominantly reflect an organism’s response to cancer, contrasting the usual goal to identify clinically relevant and even causal contributors [5]. In this article, we demonstrate that allelic dispersion (AD) is far more effective for this purpose.

**Figure 1:**
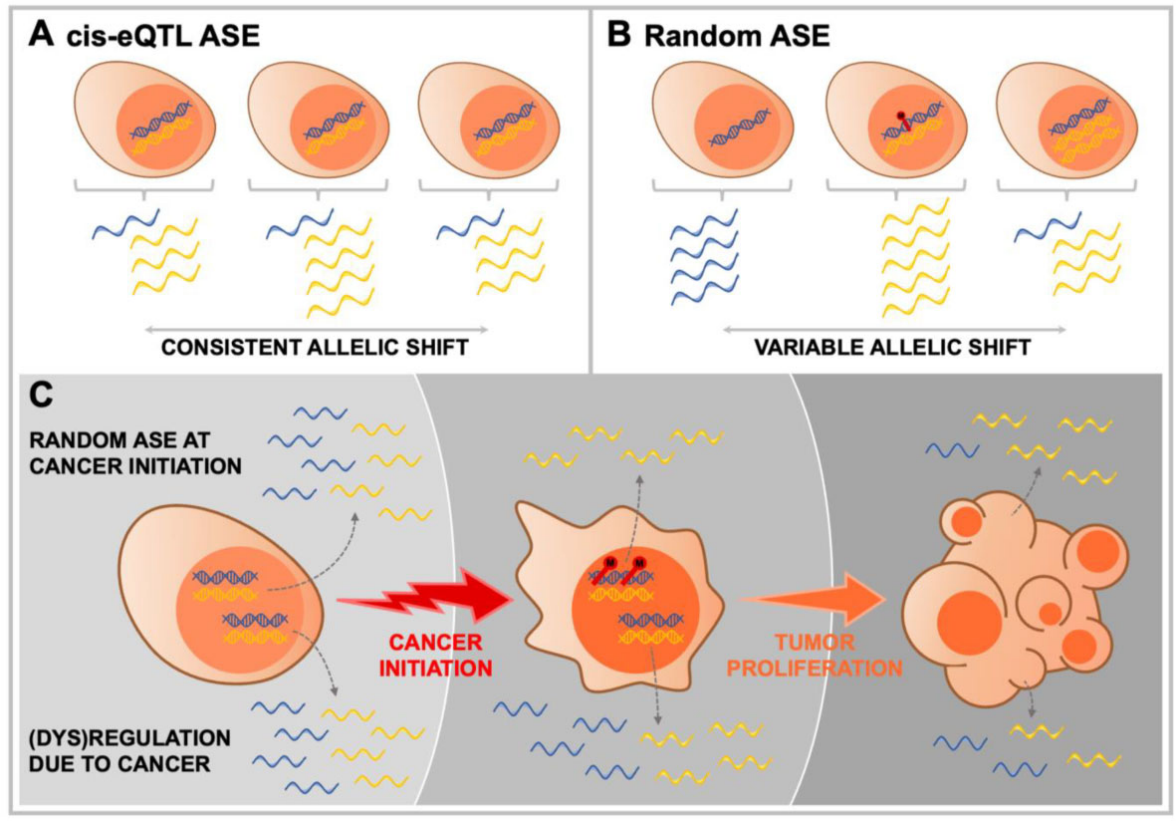
Types of ASE at the cell and tissue level. (A) For *cis*-eQTLs, expression depends on nearby genetic variation. The causes consistent expression differences between both alleles of heterozygous cells. (B) Random ASE, such as caused by (left to right) a copy number loss, DNA hypermethylation (also in the case of normal RME), and a copy number gain, can affect either allele. This causes a difference in expression (shift) between both alleles that is not consistent but variable. (C) For random ASE effects in a cell to be detectable at the tissue level, it needs to be mitotically amplified. Here, this is illustrated for cancer, where proliferation of an early cancer cell featuring allele-specific hypermethylation of a gene leads to tissue-wide hypermethylation and ASE of that gene (upper gene). Note that tumor impurity may lead to some residual biallelic expression. In contrast, for a gene directly or indirectly affected by the tumor (lower gene), both alleles will feature largely equal up- or downregulation. The upper gene’s downregulation, due to an early dysregulating event, is far more compatible with causality in cancer initiation or development than simply biallelically downregulated genes.

AD is a specific manifestation of allele-specific expression (ASE), a catch-all term for phenomena that differentially affect the expression of both copies of a gene. Available ASE methods predominantly focus on *cis*-expression quantitative trait loci (*cis*-eQTLs), that is, genetic variants that regulate the expression of nearby genes. This type of ASE is straightforward to detect, since an allele linked to a specific genetic variant will be consistently higher expressed than an allele linked to another variant for that locus (Fig. 1A). Other ASE effects do not depend on genetics. For example, genomically imprinted genes express only a single allele, which solely depends on the parent of origin, which can be exploited for their study [6]. However, many remaining ASE phenomena affect random alleles and can thus differ from cell to cell even within an individual. This includes natural random monoallelic expression (RME) and X chromosome inactivation (XCI) in females. Also, in disease, (epi)genetic aberrations such as copy number alterations or epimutations may randomly affect a single allele, causing an offset in otherwise balanced allelic expression. These types of “random” allele-specific expression do not depend on genetic variation or inheritance, which makes their study less straightforward. Given enough samples, however, affected genes will be characterized by a consistent deviation from balanced allelic expression across cells (and individuals), which we call allelic dispersion or AD (Fig. 1B).

**Figure 2:**
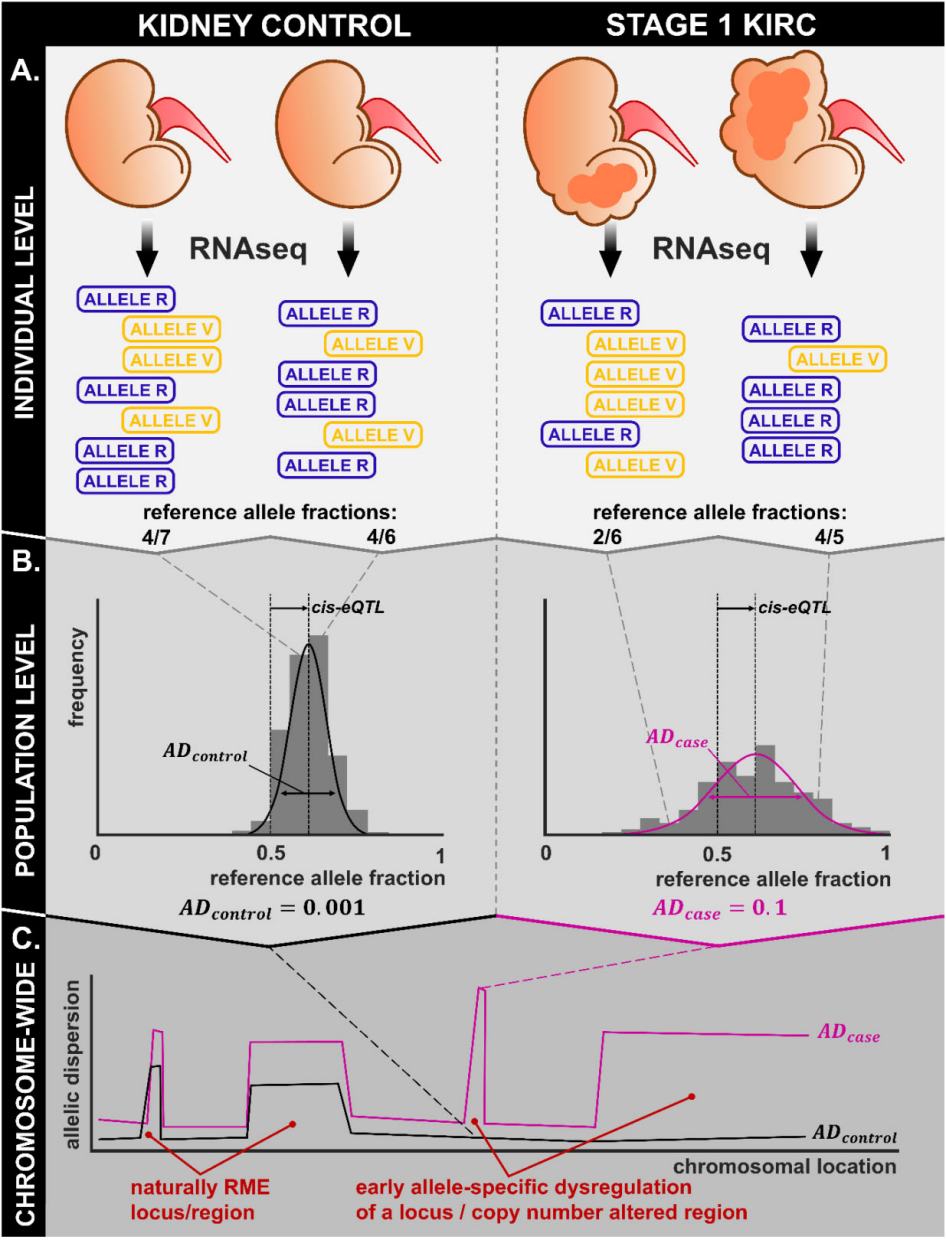
Graphical overview of *maelstRom*’s differential allelic dispersion analysis. (A) Our input consists of population-level reference and variant allele bulk RNA-seq counts for a gene (respectively, ALLELE R and V). (B) While *cis*-eQTL effects are captured by a population-consistent shift in the reference allele fraction (Fig. 1A), random ASE is captured by the latter’s variability (Fig. 1B), which we call allelic dispersion (AD). Differential (case-control) allelic dispersion (dAD) reflects disease-specific, early-occurring (Fig. 1C) ASE. (C) When applied in a genome-wide manner, (differential) AD indicates random monoallelically expressed loci in (healthy) populations, as well as early dysregulation in cancer and other diseases.

Up until now, AD has been ignored or considered a constant nuisance parameter [7, 8]. This makes sense, as it can be argued that when a single random allele is expressed in a single cell, this random effect cancels out when considering a tissue consisting of a large number of cells. However, there is one important exception to this: if a cell expresses a randomly selected allele for a gene (Fig. 1B), and the cell is mitotically expanded into a tissue, then the original ASE effect will be maintained in the resulting tissue (Fig. 1C). These exact conditions directly yield AD’s greatest use-case: the detection of allele-specific dysregulation during early carcinogenesis (Fig. 2).

Indeed, phenomena such as copy number alterations (CNAs), epimutations, and promoter mutations are all common in cancer, and they are—individually—allele-specific. They can occur in the original cancer cell targeting a single allele and be clonally maintained during cancer growth, leading to consistent differences in expression between both alleles in the resulting cancer sample (Fig. 1C, Fig. 2A). Hence, if such an allele-specific aberration frequently occurs in cases, these consistent differences will lead to an increased variance in the expression of both alleles across cases. This is detectable as differential AD (dAD) in bulk RNA-seq data of cases versus controls (Fig. 2B). In contrast, genes responding to cancer-associated dysregulation will not feature dAD. Even though such genes may exhibit DE, both alleles will be up- or downregulated to a similar extent (Fig. 1C).

Consequently, we put forward that the properties of population-level dAD-dysregulated genes—sufficiently prevalent, allele-specific, near cancer initiation—make them far better candidates to causally contribute to carcinogenesis than merely DE genes. Of note, RME (including XCI) is not disease-specific but is also associated with the expression of a single allele in the original cancer cell (as in any cell), leading to dAD upon amplification in cancer (Fig. 2C). However, autosomal RME effects are assumed to be rare [9].

In this study, we present the Modeller of Allele-Specific Transcriptomics (*maelstRom*), an R/C++ software package enabling dAD analysis, requiring solely population-level bulk RNA-seq data (Fig. 2; github.com/Biobix/maelstRom). We first introduce its methodology and our case study dataset, The Cancer Genome Atlas (TCGA) renal clear cell carcinoma cohort (KIRC). This is a cancer of metabolic origin featuring well-described CNAs (such as 3p loss) [10]. We subsequently benchmark *maelstRom* on these CNAs and simultaneously find virtually no evidence for RME beyond XCI and known loci, with the protocadherin clusters as a striking exception. Finally, dAD results unveil both known and hitherto unknown early dysregulated genes and pathways, such as GSTP1, FBP1, CCDC8, and the pentose phosphate pathway. dAD results are particularly enriched for metabolism-related genes, demonstrating that *maelstRom* indeed captures early dysregulation events in renal cancer. Moreover, a comparison with state-of-the-art literature supports a causal impact on carcinogenesis for many key dAD results.

### Implementation

*maelstRom* enables the quantification of AD and its case-control comparison, starting from per-sample allelic counts (Fig. 2A). A step-by-step analysis protocol is available online (biobix.github.io/maelstRom/articles/maelstRom_Allelic_Dispersion_tutorial), and algorithmic and mathematical details of *maelstRom*’s implementation not required to grasp the general design and applicability are described in this article’s Methods.

### *maelstRom’s* core model

**Figure 3:**
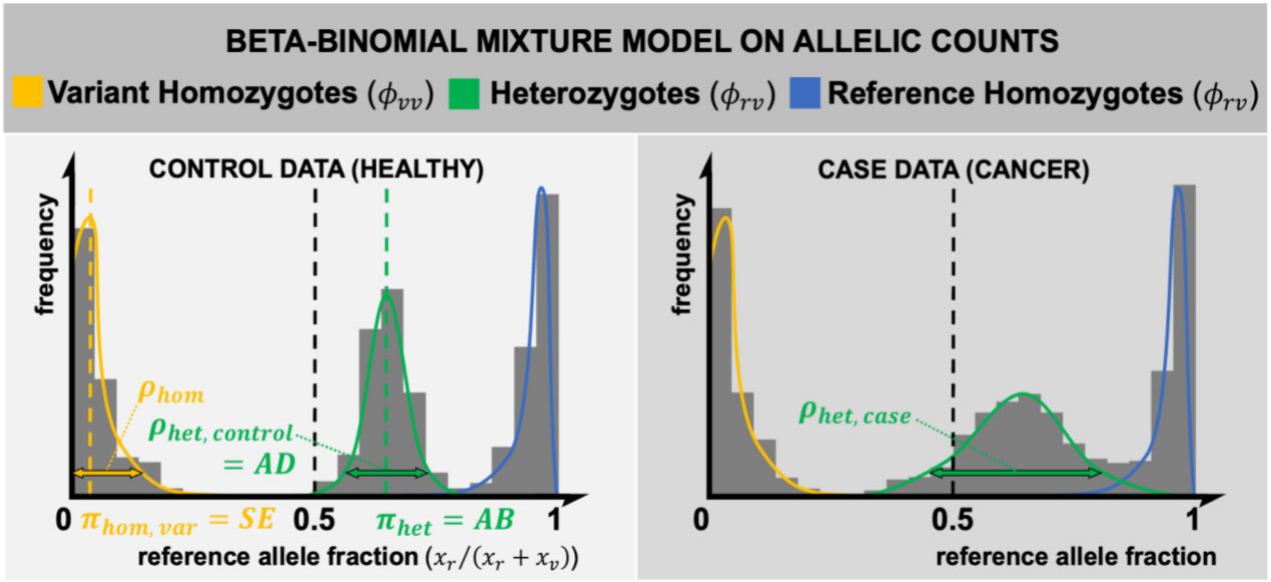
The beta-binomial mixture model for control (left) and case (right) allelic counts per SNP. On solely control data (left; Equation 1), this already allows for AB detection and AD quantification. A joint fit on case and control data (right; Equation 4), which shares all distributional parameters between cases and controls except ${{\boldsymbol{\rho }}}_{{\boldsymbol{het}},{\boldsymbol{\ \textit{control}}}}\not={{\boldsymbol{\rho }}}_{{\boldsymbol{het}},{\boldsymbol{\ \textit{case}}}}$, can test for differential AD when contrasted to Equation 1’s model fit via a likelihood ratio test.

RNA-seq can capture ASE only if it can distinguish alleles within individuals, thus requiring genetic variation. Since both alleles are hard to discern using short-read data, in practice, AD is modeled at the single-nucleotide polymorphism (SNP) level, focusing on solely heterozygous individuals. However, unlike most ASE modelers, *maelstRom* requires no genotyping data to identify these heterozygotes. Instead, ASE parameters are inferred by fitting per-SNP beta-binomial mixture models (with 1 mixture component for every possible genotype) on RNA-seq–derived allele counts through the expectation–maximization algorithm (Equation 1; Fig. 3, left):


(1) \begin{eqnarray*}
PMF\left({x}_r,{x}_v \right) &=& {\phi }_{rr}*{BetaBin}\left({x}_r|n = {x}_r + {x}_v,\,\, \pi = 1 - SE,\,\, \rho = {\rho }_{hom} \right)\\
&& + {\phi }_{rv}*{BetaBin}\left({x}_r|n = {x}_r + {x}_v,\,\, \pi = {\pi }_{het} = {\boldsymbol AB},\right.\\
&& \left.\qquad\qquad\qquad \rho = {\rho }_{het} = {\boldsymbol AD} \right)\\
&& + {\phi }_{vv}*{BetaBin}\left({x}_r|n = {x}_r + {x}_v,\,\, \pi = SE,\,\, \rho = {\rho }_{hom} \right)
\end{eqnarray*}

The beta-binomial distribution ($BetaBin$) is a straightforward extension of the regular binomial distribution, which supplements the binomial’s mean-shifting parameter ($\pi $) with a variance-increasing parameter ($\rho $). $\pi $ captures *cis*-eQTL effects in heterozygotes but can also be influenced by several technical effects, such as sequencing or alignment biases toward a certain allele. As such, we generically term this shift in mean allelic bias (AB). $\rho $, on the other hand, captures AD in heterozygotes. As for Equation 1’s remaining components (Fig. 3, left), ${x}_r$ and ${x}_v$ are per-sample allelic counts of the modeled SNP’s (arbitrarily assigned) reference and variant allele. ${\phi }_{rr}$, ${\phi }_{rv}$, and ${\phi }_{vv}$ reflect the population frequencies of this allele’s reference homozygotes, heterozygotes, and variant homozygotes. $SE$ indicates sequencing (and other technical) errors and, as such, dictates the $\pi $ parameter in homozygous samples. Finally, ${\rho }_{hom}$ reflects extra variance in homozygotes but is, unlike ${\rho }_{het}$, a mere nuisance parameter. For simplicity, we further refer to the informative ${\rho }_{het}$ as $\rho $ in this article.

Note that modeling AD as a beta-binomial variability parameter (known as the overdispersion parameter) inherently accounts for sample coverage differences (through its *n* parameter; Equation 1). Simply estimating AD as the observed variability of (reference) allele fractions would lead to biased results since low-count derived fractions are intrinsically more variable.

### Metaparameter estimation

Among Equation 1’s components, $SE$ is the only population-level metaparameter and is thus robustly estimated before assessing (d)AD. *maelstRom* achieves this by fitting a grossly simplified version of Equation 1 to all available per-SNP allele counts and calculating its median $SE$ value across SNPs (Equation 2):


(2) \begin{eqnarray*}
PMF \left({x}_r,{x}_v \right) &=& {\phi }_{rr}*{Binomial}\left({x}_r|n = {x}_r + {x}_v,\,\, p = 1 - SE \right)\\
&& + {\phi }_{rv}*{Binomial}\left( {{x}_r|n = {x}_r + {x}_v,\ p = 0.5} \right)\\
&& + {\phi }_{vv}*{Binomial}\left( {{x}_r|n = {x}_r + {x}_v,\ p = SE} \right)
\end{eqnarray*}

In a similar vein, the population’s inbreeding coefficient (${F}_{\textit{inbr}}$) is estimated as its across-SNP median from Equation 2’s genotype frequencies, for later use in Hardy–Weinberg equilibrium (HWE)–based quality filtering of *maelstRom*’s results:


(3) \begin{eqnarray*}
{F}_{inbr} &=& \frac{{observed\,\, heterozygosity}}{{expected\,\,hetezozygosity\,\,assuming\,\, panmixis}}\\
&& = 1 - \frac{{\phi }_{rv}}{2 *\left({\phi }_{rr} + {\phi }_{rv}/2 \right)*\left({\phi }_{vv} + {\phi }_{rv}/2 \right)}
\end{eqnarray*}

Though both $SE$ and ${F}_{\textit{inbr}}$ can be set through knowledge of the used sequencing technology and the population under study (e.g., often panmixis can be assumed), we recommend their estimation, as setting these parameters overly strict can hamper *maelstRom*’s later model fit. Equation 2’s fit is also extremely quick, as analytical solutions exist for binomial parameter (maximum likelihood) estimates, unlike Equation 1 (see Methods).

### Differential allelic dispersion fit

To detect differential AD in a case-control scenario (Fig. 2B, Fig. 3), Equation 1 is extended by indicator variables, ${I}_{\textit{control}}$ and ${I}_{\textit{case}}$, to designate the subpopulation per sample:


(4) \begin{eqnarray*}
PMF\left({x}_r,{x}_v \right) &=& {\phi }_{rr}*{BetaBin}\left({x}_r|n = {x}_r + {x}_v,\,\, \pi = 1 - SE,\,\, \rho = {\rho }_{hom} \right)\\
&& + {\phi }_{rv}*{BetaBin}\left({x}_r|n = {x}_r + {x}_v,\,\, \pi = {\pi }_{het},\right.\\
&& \left. \qquad\qquad\qquad \rho = {I}_{control}{\rho }_{het,control} + {I}_{case}{\rho }_{het,case} \right)\\
&& + {\phi }_{vv}*{BetaBin}\left({x}_r|n = {x}_r + {x}_v,\,\, \pi = SE,\,\, \rho = {\rho }_{hom} \right)
\end{eqnarray*}

Fitting both Equations 1 and 4 on an SNP’s per-sample allele counts allows this SNP to be tested for dAD using a likelihood ratio test (LRT) with 1 degree of freedom. To achieve both accurate and fast results, *maelstRom* employs—among others—numerical starting estimates of both ${\pi }_{het}$ and ${\rho }_{het}$ (Equation 1) and ${\rho }_{het,\textit{control}}$, ${\rho }_{het,\textit{case}}$ (Equation 4) derived from Kleinman’s work [11], custom C++ implementations of the beta-binomial density and its parameters’ numerical gradients and estimation (the former are used in the latter), and sample outlier detection and correction through Cook’s robust sample deletion procedure [12]. Our Methods extensively cover these implementation details.

### SNP-to-gene combination

While SNP-level dAD results can be explored directly, results at the gene or transcript level are more biologically interpretable. Many ASE studies address this through direct gene-level modeling [13, 14] or (weighted) *P* value averages across SNPs per gene [15]. The first option requires genotyping-dependent allele-specific transcript assemblies; the second has no clear statistical interpretation and can, additionally, not consolidate independent evidence of multiple SNPs into 1 stronger gene-level conclusion (an average cannot be more extreme than any of its component values). As these would, respectively, nullify *maelstRom*’s minimal data and preprocessing requirements (i.e., only RNA-seq) and statistical rigor, we instead implemented Dai et al.’s [16] dependence-aware modified Lancaster method, which estimates gene-level combined *P* values through an approximately ${\chi }^2$-distributed statistic ${T}_{\textit{ModLan}}$ (Equation 5):


(5) \begin{eqnarray*}
{T}_{ModLan} &=& c*{T}_{Lan}\\
&& = c*\sum\nolimits_{i = 1}^N {\gamma _{\left( {{w}_i/2,\ 2} \right)}^{ - 1}\left( {1 - {p}_i} \right) \approx \chi _v^2\quad {when\,\,{H}_0\,\,is\,\,TRUE}}
\end{eqnarray*}

In Equation 5, ${p}_i$ are the separate SNP-level dAD *P* values corresponding to the same gene, and ${w}_i$ are SNP-level weights denoting their relative contribution to the combined *P* value (here the root of its median coverage times heterozygote frequency). Both *c* and *v* are constants that reflect the dependency between SNPs, which increases the combined *P* value if present. After all, allele counts of SNPs that occur very proximally along a gene’s (processed) mRNA are very likely to be derived from the same RNA-seq read or even mRNA molecule, in which case, combining them as statistically independent evidence could produce false-positive statistical artifacts.

*c* and *v* must both be estimated through random iterative shuffling of sample labels (Equation 4: ${I}_{\textit{control}}$ and ${I}_{\textit{case}}$) and redoing the entire dAD analysis. Using *maelstRom*’s default of 10,000 iterations, this would be overly computationally intensive. We thus extended Dai et al.’s [16] method through incorporating the score test (instead of our regular LRT) when calculating iterative dAD *P* values. The score test’s required model fit is independent of sample label shuffling (${I}_{\textit{control}}$ and ${I}_{\textit{case}}$) and can thus be reused across iterations. The score test’s drawback of only providing *P* values without parameter estimates is of no concern here, as only *P* values are required for SNP dependence correction. It is worth noting that this strategy could be reused in future studies that seek to combine SNP-level results with gene-level data if minimal data preprocessing or assumptions are desired. The Methods section contains derivations for *c* and *v* (and also ${w}_i$).

### Canonical dAD

**Figure 4:**
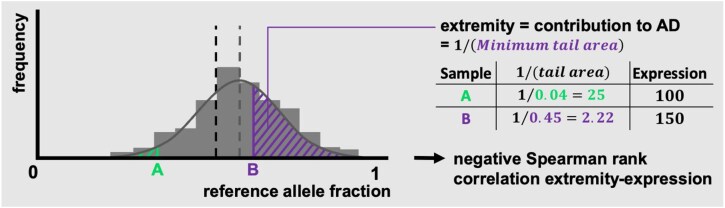
Evaluating canonical dAD. Here, a gene is less expressed in sample A than in sample B, exhibiting an expression count of 100 versus 150. Sample A similarly contributes more to the gene’s AD, as it exhibits a relatively extreme difference in expression between both alleles. Given the population’s beta-binomial fit, this “extremity” is, mathematically, inversely proportional to a sample’s minimal tail area. Here, this is the left tail for sample A (green) and the right tail for sample B (purple). Thus, ${{\boldsymbol{p}}}_{{\boldsymbol{canon}}}$ is obtained by a standard Spearman rank correlation test on each sample’s (inverse) minimal tail area and its expression. In this figure’s example, increased AD has a negative impact on expression, suggesting an expression downregulation allele-specific event.

For dAD to actually reflect biological impact, it ideally co-occurs with DE at the sample level—for example, early-occurring promoter hypermethylation leading to expression downregulation, or copy number loss or gains leading to, respectively, expression down- or upregulation in the affected sample. Extending this reasoning to the population level, one would expect the samples with the highest prevalence of (copy number, hypermethylation, etc.) aberrations to contribute most to the increased AD (i.e., show most skewed allelic expression, Fig. 4 observation A) and simultaneously contribute most to DE (i.e., show relatively high or low expression). To assess this, we developed a “canonical dAD” test, which is a Spearman rank correlation test between the aforementioned sample-level contributions to dAD and DE, yielding ${p}_{\textit{canon}}$ per SNP (Fig. 4, Methods).

It should be noted that ${p}_{\textit{canon}}$ is intended to, exploratively, indicate the most biologically relevant dAD results and should not be considered a hard filter. A first reason is that only heterozygote samples can contribute to this test, leading to inherently low power. Additionally, it cannot account for complex dAD(-interfering) patterns, such as double epigenetic hits on both alleles or additional expression modulation on top of dAD in either the actual tumor cells or the tumor’s environment. Hence, the Results section employs ${p}_{\textit{canon}}$ when identifying individual genes featuring the most overt allele-specific dysregulation during early carcinogenesis but not during subsequent (gene set) overrepresentation analyses. Furthermore, ${p}_{\textit{canon}}$ is used liberally: for every gene, the most significant ${p}_{\textit{canon}}$ among its SNPs is reported (Family-Wise-Error-Rate-corrected across SNPs for that gene; see Methods).

### Case study data acquisition and preprocessing

For the case study, we used TCGA’s renal clear cell carcinoma (KIRC) cohort, which provides 72 control and 268 stage 1 tumor (case) RNA-seq BAM files via the GDC data portal [17]. BAM files were aligned to human reference genome GRCh38. Repeatedly analyzed individuals were deduplicated by retaining the most recent sample BAM file. Only stage 1 cases were used as these are closest to tumor initiation, which is in line with our aim of finding early dysregulation. We subsequently used mpileup/bcftools from SAMtools [18] to infer SNP allele counts from BAM files, after indexing if necessary, retaining only those with a minimal raw read depth of 10 in at least 1 sample, and listed in the dbSNP database [19]. We filtered out nonuniquely mapped reads to reduce noise. For X chromosomal SNPs, only XX female samples were considered (consisting of 20 controls and 105 cases), as XY males cannot be heterozygous for these SNPs.

As *maelstRom*’s beta-binomial models (Equations 1 and 4) permit only 2 alleles, the 2 most common dbSNP-listed alleles (based on summed allele counts across all samples) were considered for dAD analysis and termed “reference” and “variant” allele correspondingly. Only SNPs supported by nonzero reference or variant allele counts in at least 10 samples were retained, leading to 127,023 autosomal and 2,083 remaining X chromosomal SNPs. These were used as input for *maelstRom*’s above-described dAD analysis up to, and including, SNP-to-gene combination (using SNP annotation provided by dbSNP [19]). Together with basic quality, goodness-of-fit-, and annotation filters (minimum required median coverage and number of heterozygotes, no unrealistic *maelstrom* parameter estimates, HWE conformity, SNPs having a genetic annotation, and the gene being present in DE analysis results based on gene-level htseq count files provided by Xenabrowser [20]: see Methods for full details) delivered final dAD results for 11,325 autosomal and 291 X chromosome genes.

Finally, CNA occurrence and DE results data were obtained from Xenabrowser [20] (using their htseq gene count file and gistic2 thresholded gene-level CNA data) and Infinium Humanmethylation450 array probe data from MEXPRESS [21]. All of these were analyzed using standard established pipelines (*EdgeR* for DE, *fisher.test* from the *stats* R package for promoter hypermethylation, and CNA occurrence simply used as such), and the Methods section provides more details.

## Results

### *maelstRom* benchmarking through known CNA and RME

**Figure 5:**
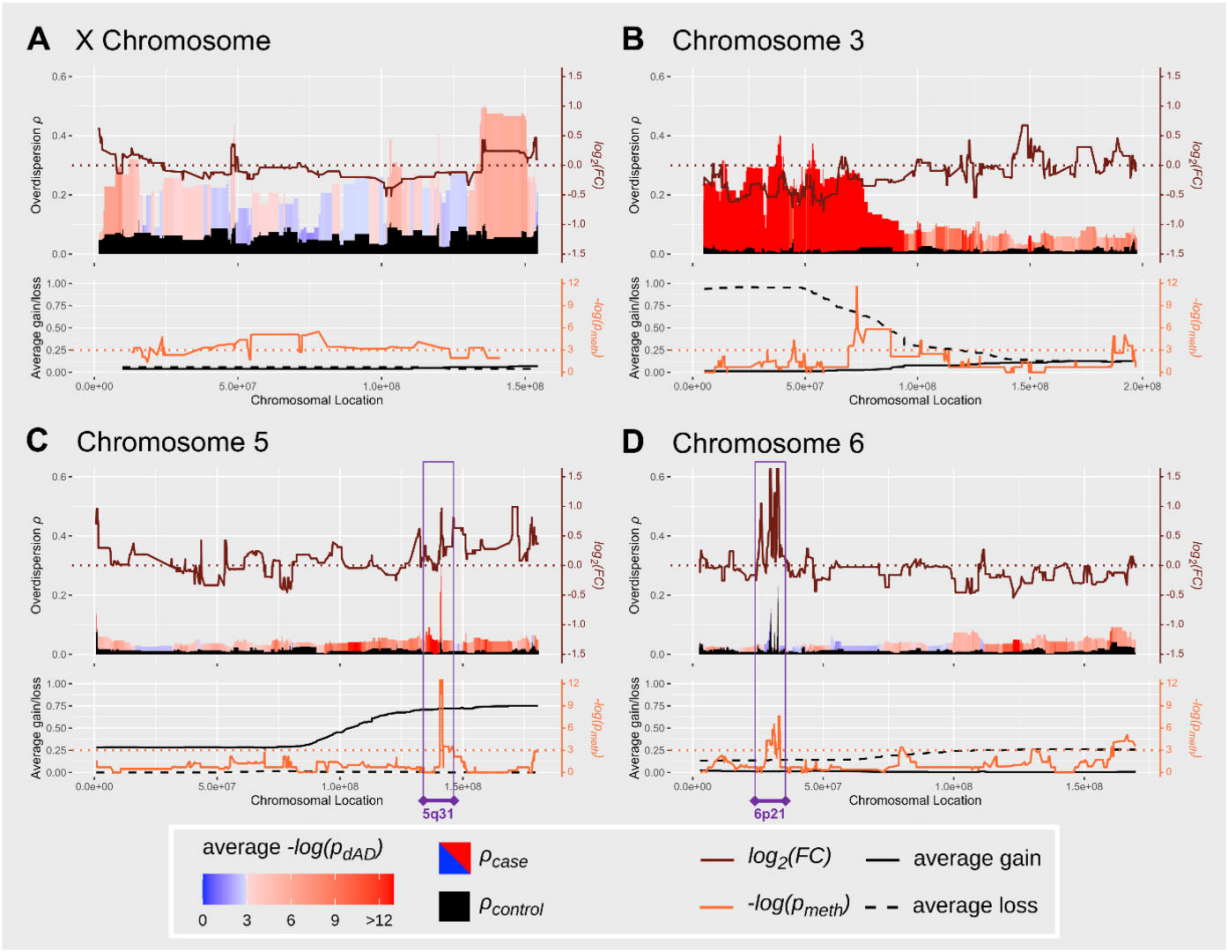
Chromosome-wide gene-level (d)AD results for TCGA KIRC data, as ${\boldsymbol{\rho }}$, for chromosomes X (A), 3 (B), 5 (C) and 6 (D). Top plots within each panel display ${{\boldsymbol{\rho }}}_{{\boldsymbol{control}}}$ in black and ${{\boldsymbol{\rho }}}_{{\boldsymbol{case}}}$ color-coded according to statistical significance of differential AD (FDR-corrected ${{\boldsymbol{p}}}_{{\boldsymbol{dAD}}}$; turns from blue to red at 0.05), together with DE results as ${\boldsymbol{lo}}{{\boldsymbol{g}}}_2( {{\boldsymbol{Fold\ \textit{Change}}}} )$. Bottom panels display *P* values testing for tumor promoter hypermethylation ($- {\boldsymbol{log}}( {{{\boldsymbol{p}}}_{{\boldsymbol{meth}}}} );$ raw *P* value with orange dotted line at ${{\boldsymbol{p}}}_{{\boldsymbol{meth}}}$ = 0.05) and the average copy number loss and gain in tumor samples. All measures are visualized as rolling medians (window size of 15 genes) to emphasize genomic regions rather than individual genes; some regions of interest are highlighted by purple windows. dAD significance is relatively low across the X chromosome as these analyses solely rely on female samples, drastically reducing the number of controls (72 to 20) and cases (268 to 105).

**Figure 6:**
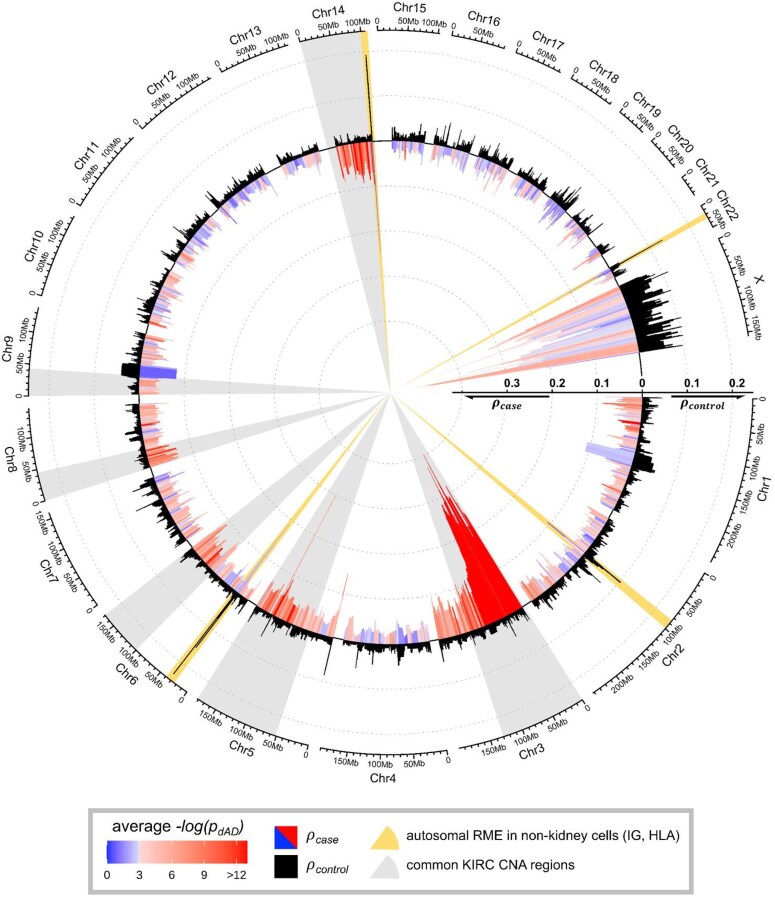
Genome-wide gene-level (d)AD results for TCGA KIRC data, as ${\boldsymbol{\rho }}$. Similar to Fig. 5’s top plots, ${{\boldsymbol{\rho }}}_{{\boldsymbol{control}}}$ is displayed in black, ${{\boldsymbol{\rho }}}_{{\boldsymbol{case}}}$ is color-coded according to statistical significance of differential AD (FDR-corrected ${{\boldsymbol{p}}}_{{\boldsymbol{dAD}}}$; turns from blue to red at 0.05), and both are visualized as rolling medians (window size of 15 genes) to emphasize genomic regions rather than individual genes. Regions of interest are highlighted as colored sections. Autosomal RME whose expression is of nonkidney origin is colored yellow; note the high AD in both cases and controls, as well as the nonsignificant dAD. Common CNA regions in KIRC are colored gray (5q, 6q, 8p, 9p, and 14q); note their significantly increased AD in cases compared to controls across large genomic regions.

Applying *maelstRom* on TCGA KIRC cancer-control data yields (d)AD results for 11,325 autosomal genes, of which 2,142 show significant dAD with a relevant effect size (1E-3 false discovery rate [FDR], ${\rho }_{\textit{case}} > 1.5\ {\rho }_{\textit{control}}$; Supplementary Data 1). Figure 5 displays these results in detail across 4 chromosomes of particular interest, together with potentially underlying (epi)genetic events (promoter hypermethylation and CNAs). Genome-wide (d)AD results are depicted as a circos plot [22], indicating multiple genomic regions with aberrant AD patterns (indicative of CNA and RME events) (Fig. 6). In general, these figures depict low AD in controls (mean ${\rho }_{\ \textit{control}}$ = 0.029) and increased AD in cancer (mean ${\rho }_{\textit{case}}$ = 0.053).

For benchmarking, we first considered the X chromosome (291 genes). As XCI occurs during early embryonal development and is typically skewed toward 1 chromosome in 1 individual female [23], a higher AD is already expected in controls and here observed (mean ${\rho }_{\textit{control}}$ of 0.091). Moreover, in KIRC, clonal amplification of the original cancer cell with 1 silenced X chromosome leads to a tumor mass (largely) consisting of cells with the same silenced chromosome. Hence, this should lead to increased AD for the X chromosome, which we observe for female cancer samples (mean ${\rho }_{\textit{case}}$ of 0.264). We also evaluated KIRC’s hallmark 3p arm loss, occurring in >90% of cases [10]. This region features unremarkable AD in controls. However, 3p loss of (mostly) a single allele in the original tumor cell leads to extreme dAD across chromosome 3’s p-arm upon clonal amplification (Fig. 5B). Similarly, other common CNA events are characterized by consistently elevated AD in cancer (5q, 6q, 8p, 9p, and 14q; Figs 5 and 6; Supplementary Figs. S1–S4 contain TCGA-provided CNA occurrence for proof). Importantly, even in samples subject to 3p loss, expression is not expected to be entirely monoallelic due to tumor impurity. Particularly for genes whose expression is low in cancer cells compared to infiltrating and stromal cells, the observed dAD effect size will be less pronounced in bulk RNA-seq data.

Four other gene clusters exhibit very high AD in control tissue: HLA (6p21; Fig. 5D, Fig. 6) and 3 immunoglobulin clusters IGKV, IGHV, and IGLV (immunoglobin kappa, heavy, and light variable chains; 2p12, 14q32, and 22q11; Fig. 6). These clusters are known to feature RME but are expressed in leukocytes and not in kidney cells [24, 25], explaining their general lack of differential AD, despite their naturally high AD and strong expression upregulation in cancer as part of the adaptive immune response (Fig. 5D, brown line).

Together, these results demonstrate that *maelstRom*’s AD analysis is able to identify nongenetically determined ASE effects, induced by clonal amplification (XCI’s already high control AD and its increase in cancer, 3p loss, and other CNAs in KIRC). Naturally occurring RME loci also feature a high control AD, but their expression is here of nonkidney origin (HLA and IG clusters) and thus not clonally amplified in cancer (no dAD).

### Autosomal RME is not widespread

The X chromosome’s AD increase in kidney cancer illustrates that the presence of RME in a normal epithelial cell will be detected as dAD when clonally amplified in cancer (Fig. 1C). Hence, RME is a potential source of false positives when studying cancer-specific effects. We therefore identified those significant dAD genes compatible with RME as observed for the X chromosome, meaning that they should exceed the X chromosome’s mean AD in both cases and controls (${\rho }_{\textit{control}} \ge $0.091, ${\rho }_{\textit{case}} \ge $0.264). When considering the 6,721 autosomal genes covered by at least 2 SNPs, only 9 genes met these criteria, with only 3—PCDHGB7, IGHG1, and CYP4F11—not located on the 3p arm.

Of these, IGHG1 is already known to feature RME in leukocytes (see benchmarking section) but is here—as an outlier among those genes—also detected as dAD. CYP4F11 encodes a less characterized cytochrome P450 enzyme expressed in kidney [26], without prior knowledge regarding RME status. Most interestingly, PCDHGB7 is a clustered protocadherin (PCDH), which occur as $\alpha $-, $\beta $-, and $\gamma $-clusters at 5q31. In Purkinje neurons, stochastic (thus low) and random monoallelic expression has been extensively described for clustered PCDHs, with the exception of C-type $\gamma $-PCDHs [27, 28]. In kidney cancer, most clustered PCDHs feature low expression (and were therefore initially filtered) but similarly high (d)AD, with the exception of a biallelic, highly expressed C-type $\gamma $-PCDH (Supplementary Table S1). This similarity clearly supports clustered PCDHs as RME candidates in kidney. However, also frequent promoter DNA hypermethylation and 5q copy number gains are observed for this locus in KIRC (Fig. 5C). Hence, additional experimental validation is required to confirm RME for this gene cluster (as well as CYP4F11) in kidney.

Overall, it is clear that the number of loci with (d)AD statistics compatible with RME is extremely low, aligning with the state-of-the-art regarding the prevalence of autosomal RME effects beyond well-established cases [9, 29].

### *maelstRom* identifies early allele-specific aberrations in renal clear cell carcinoma

After benchmarking and demonstrating a minimal impact of RME, we aimed at identifying individual genes featuring major early allele-specific dysregulation in KIRC. We therefore excluded major KIRC’s major CNA regions (see benchmarking section) and imposed a more stringent significance cutoff (FDR <1E-10). Moreover, as an additional safeguard against RME and technical artifacts, we solely considered genes with significant DE between cases and controls, as well as showcasing “canonical” dAD (see Methods). These criteria retained the 17 top genes, presented in Table 1A. Biological relevance of obtained results in (renal) cancer is greatly supported by pertinent studies (*Ref* column) describing functional validation of the causal impact for a subset of presented results.

**Table 1:** Key dAD genes, both at the gene level (A) and among dAD-enriched GO terms (B), sorted by decreasing ${{\boldsymbol{\rho }}}_{{\boldsymbol{case}}}$. Results are presented for dAD (ρ_control_, ρ_case_, FDR-adjusted p_dAD_) and DE (log_2_FC, FDR-adjusted p_DE_) analysis and canonical dAD assessment (${\boldsymbol{Cor}}{{\boldsymbol{r}}}_{{\boldsymbol{canon}}}$; ${{\boldsymbol{p}}}_{{\boldsymbol{canon}}}$ see Implementation and Methods sections). The final 2 columns (Rating, Ref) provide state-of-the-art evidence for the listed genes’ relevance in (renal) cancer, being ranked as follows. ****: Functional experiments demonstrating causality in renal cancer. ***: Functional experiments demonstrating causality pan-cancer/in other cancer types. **: Biomarker value in renal cancer. *: Strong indication that the gene (or its dysregulation) is generally involved in carcinogenesis (e.g., through apoptosis, angiogenesis). –: Indirect links to carcinogenesis, either through a gene paralog (ALPK3) or antisense protein (NCBP2).

Gene	ρ_control_	ρ_case_	p_dAD_	log_2_FC	p_DE_	Corr_canon_	p_canon_	Rating	Ref
**A**. Top dAD genes with relevant Corr_AD-EX_ (same sign as log_2_FC; p_AD-EX_ < 0.05)									
CCDC8	0.002	0.264	8.55E-15	−1.139	2.01E-08	−0.371	1.72E-02	***	Dai et al. *Proc Natl Acad Sci. USA* 2011 [30] Zhang et al. *Cancer Med*. 2021 [31] Morris et al. *Oncogene*. 2011 [32]
ALPK3	0.048	0.177	3.30E-12	0.650	3.27E-04	0.241	2.80E-02	−	Jiang et al. *Exp Cell Res*. 2020 [54]
TMC4	0.008	0.163	4.49E-18	−1.986	5.18E-20	−0.492	1.40E-03	**	Song et al. *Front Immunol*. 2021 [48] Tang et al. *Sci Rep*. 2023 [49]
AMOTL2	0.009	0.160	1.25E-17	−0.182	7.74E-02	−0.388	9.72E-05	*	Guo et al. *Mol Ther Nucleic Acids*. 2020 [38] Mojallal et al. *Nat Commun*. 2014 [39]
ECHS1	0.004	0.140	5.50E-15	−1.326	2.26E-37	−0.417	7.86E-05	****	Qu et al. *Cancer Res*. 2020 [57]
HAGLR	0.009	0.139	1.83E-15	−0.786	9.88E-09	−0.410	1.32E-02	***	Chan and Tay *Int J Mol Sci*. 2018 [43] Wang et al. *Mol Cancer*. 2017 [44]
CLDN7	0.005	0.111	2.68E-17	−1.092	2.44E-09	−0.229	2.70E-02	****	Li et al. *J Exp Clin Cancer Res*. 2018 [34]
CTSF	0.005	0.103	9.87E-14	−0.240	2.57E-02	−0.288	3.60E-03	****	Zhou et al. *Sci Rep*. 2024 [35]
MLF1	0.010	0.100	2.42E-15	−0.846	8.74E-16	−0.363	9.87E-04	***	Yoneda-Kato et al. *EMBO J*. 2005 [36] Yoneda-Kato and Kato *Mol Cell Biol*. 2008 [37]
BSPRY	0.001	0.100	4.54E-12	−1.854	8.09E-23	−0.471	3.75E-03	**	Yang et al. *J Cancer Res Clin Oncol*. 2023 [50] Bin Satter et al. *Cancers (Basel)*. 2022 [51] Bret et al. *Oncotarget*. 2012 [52] Kohn et al. *PLoS One*. 2014 [53]
GSTP1	0.008	0.098	1.11E-13	−0.545	2.15E-05	−0.233	4.88E-02	***	Cairns et al. *Clin Cancer Res*. 2001 [45] Millar et al. *Oncogene*. 1999 [46] Hoque et al. *Cancer Res*. 2004 [58] Louie et al. *Cell Chem Biol*. 2016 [47]
SCIN	0.007	0.085	1.35E-19	−1.875	2.25E-15	−0.479	2.11E-04	***	Zunino et al. *Blood*. 2001 [42]
DSP	0.003	0.068	2.26E-21	−1.358	7.00E-15	−0.397	1.84E-03	***	Dusek and Attardi *Nat Rev Cancer*. 2011 [41]
NCBP2	0.003	0.064	3.26E-19	−0.200	9.63E-06	−0.246	4.59E-02	−	Kugeratsk et al. *Sci Signal*. 2019 [40]
OBSL1	0.009	0.062	2.62E-13	−0.098	4.98E-01	−0.315	4.89E-02	*	Yan et al. *Mol. Cell* 2014 [33]
FBP1	0.012	0.061	2.93E-14	−2.128	7.10E-33	−0.323	1.64E-03	****	Bo et al. *Nature*. 2014 [56]
OPA1	0.009	0.055	4.57E-11	−0.430	4.31E-13	−0.282	3.29E-02	*	Herkenne and Scorrano *Aging*. 2020 [55]
**B**. Genes of interest among dAD-enriched GO terms									
PGD	0.001	0.033	1.64E-08	−0.825	3.45E-24	0.058	1.00E+00	*	Patra and Hay *Trends Biochem Sci*. 2014 [59]
H6PD	0.020	0.041	1.09E-04	0.774	7.28E-20	−0.320	9.00E-02	*	Patra and Hay *Trends Biochem Sci*. 2014 [59]
RPE	0.002	0.033	1.81E-04	−0.300	1.86E-09	−0.010	9.24E-01	*	Patra and Hay *Trends Biochem Sci*. 2014 [59]
TKT	0.009	0.318	1.35E-19	0.117	2.11E-01	1.000	8.33E-01	*	Patra and Hay *Trends Biochem Sci*. 2014 [59]
MRPS2	0.017	0.084	4.77E-04	−0.020	7.81E-01	−0.278	2.85E-03	*	Huang et al. *Int J Mol Sci*. 2020 [60]
MRPS10	0.040	0.106	3.07E-16	−0.513	2.61E-19	0.306	1.23E-03	*	Huang et al. *Int J Mol Sci*. 2020 [60]

**Figure 7:**
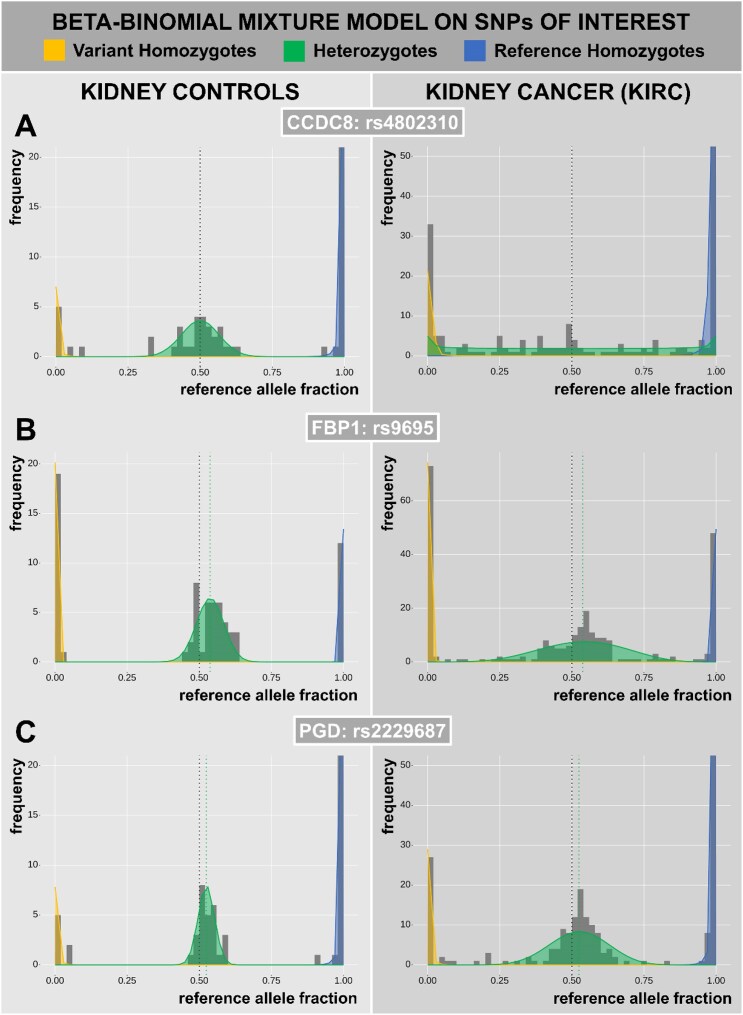
SNP-level *maelstRom* results for selected SNPs of 3 genes of interest: rs4802310 of CCDC8 (A; ${{\boldsymbol{\rho }}}_{{\boldsymbol{het}},{\boldsymbol{control}}} = $ 9.85E-6, ${{\boldsymbol{\rho }}}_{{\boldsymbol{het}},{\boldsymbol{case}}} = $ 0.365), rs9695 of FBP1 (B; ${{\boldsymbol{\rho }}}_{{\boldsymbol{het}},{\boldsymbol{control}}} = $ 7.69E-3, ${{\boldsymbol{\rho }}}_{{\boldsymbol{het}},{\boldsymbol{case}}} = $ 7.68E-2), and rs2229687 of PGD (C; ${{\boldsymbol{\rho }}}_{{\boldsymbol{het}},{\boldsymbol{control}}} = $ 3.22E-9, ${{\boldsymbol{\rho }}}_{{\boldsymbol{het}},{\boldsymbol{case}}} = $ 3.33E-2). Values of ${{\boldsymbol{\rho }}}_{{\boldsymbol{het}}}$ are given for visual comparison, with higher values generally corresponding to “broader” heterozygous peaks (but also depends on ${\boldsymbol{n}}$; cf. Equation 1).

The gene with the highest ${\rho }_{\textit{case}}$, CCDC8 (Fig. 7A, Table 1), encodes a cofactor required for p53-mediated apoptosis [30] whose downregulation is an unfavorable prognostic biomarker [31] and whose knockdown directly promotes tumor growth [32]. It is also 1 of 3 components of the genome integrity-preserving 3 M complex together with the top dAD gene, OBSL1 [33]. These results indicate the 3 M complex as frequently dysregulated in KIRC carcinogenesis and incentivize further research. In renal cancer, CLDN7’s downregulation due to promotor hypermethylation is associated with poor prognosis. Conversely, its re-(over)expression attenuates renal tumor proliferation and induces apoptosis [34]. CTSF’s causal role in renal cancer is similarly functionally supported [35]. Supporting these causality claims, both CLDN7 and CTSF are found here early dysregulated in KIRC (Table 1). Several top genes pertain to general tumor-suppressing or tumor-promoting phenomena, with their dysregulation increasing susceptibility to tumorigenesis. These include MLF1 for p53-mediated apoptosis [36, 37] and AMOTL2 [38, 39] and NCBP2 [40] for hypoxia-induced angiogenesis. In line with its strong dAD result, desmosome dysregulation (including the obligate desmosome protein DSP) has been identified as a general early and causal event in cancer through murine experiments [41].

For several remaining top genes, functional validation pinpointed a causal role in carcinogenesis, but in specific cancers other than KIRC (SCIN [42], HAGLR [43, 44], GSTP1 [45–47]). For other top results, a causal role in carcinogenesis has not been demonstrated, though they have been established as clinically relevant biomarkers (e.g., with prognostic value, such as TMC4) [48, 49]. Similarly, BSPRY has been a top predictive gene in at least 4 independent risk models and correlation-based prognostic tools in renal [50, 51] and other cancers [52, 53]^.^ Least supported in literature is the relatively uncharacterized ALPK3, with only its paralog, ALPK2, indicated as a tumor promotor in renal cancer [54]. Our results hint at a similar role for ALPK3, as *maelstRom*’s only expression-upregulated top hit (Table 1). Combined, these results provide further incentive to evaluate these genes’ causal impact in renal cancer.

Finally, ECHS1, FBP1 (Fig. 7B), and OPA1 (through its mitochondrial function [55]) all pertain to metabolism. The causal impact of the former two in renal carcinogenesis is supported by strong functional evidence: FBP1 through its central catalytic role in gluconeogenesis [56] and ECHS1 through lipid accumulation, which is of particular interest to KIRC [57]. While metabolic dysregulation commonly occurs in cancer (cf. the ubiquitous Warburg effect) [57], renal cancer shows an especially egregious metabolic shift [10], illustrated by these individual genes. Nevertheless, as metabolic pathways rely on the concerted activity of many genes, individual genes may be dysregulated in only a part of the population. As these will exhibit less pronounced population-level dAD, we also performed gene set analyses of (less stringently filtered) dAD genes.

### dAD but not DE reflects metabolic dysregulation of KIRC

We performed overrepresentation analyses (ORAs) on gene sets featuring significant DE, dAD, and both DE and dAD in TCGA KIRC (see Methods), obtaining enriched Gene Ontology (GO) terms regarding biological process (BP), cellular component (CC), and molecular function (MF). Unsurprisingly (Fig. 1C), DE genes lead to GO terms that are predominantly the consequence of cancer: “adaptive immune system” for BP, “cell surface” for CC, and “antigen binding” for MF (Supplementary  Tables S2–S4). dAD genes, however—combined with DE or by themselves—mostly return metabolic GO terms. The latter largely relate to energy, hypoxia, and organic and fatty acid metabolism (BP; Table 2), as well as oxidoreductase and lyase activity (MF; Table S4). Of interest, “generation of precursor metabolites and energy” (Table 2) includes the pentose phosphate pathway, of which 2 (out of 3) oxidative phase enzymes (PGD and H6PD; Fig. 7C) and 2 (out of 4) nonoxidative phase enzymes (RPE and TKT) feature dAD (Table 1B), but not always DE. Combined with our results for FBP1 [56], dAD analysis is far more effective than DE at indicating metabolic dysregulation as key for early KIRC development.

**Table 2:** Overrepresented biological process (BP) Gene Ontology terms among differential AD and DE genes. Resulting GO terms were filtered on redundancy, excluding terms if their involved genes showed at least 60% overlap with a more significant GO term; significance and other filter criteria are listed in Methods.

**A. BP GO-terms enriched for significant dAD (2142/11325 genes)**	
**Description**	***P*_FDR_**
Generation of precursor metabolites and energy	3.58E-07
Organic acid catabolic process	2.09E-06
Cellular amino acid metabolic process	2.20E-05
Neutrophil-mediated immunity	3.02E-03
Cellular modified amino acid metabolic process	3.18E-03
Cellular response to hypoxia	3.18E-03
Protein folding	3.18E-03
Translational termination	4.86E-03
**B. BP GO terms enriched for significant dAD and DE (942/11,325 genes)**	
Organic acid catabolic process	4.56E-16
Small-molecule biosynthetic process	1.01E-08
Fatty acid metabolic process	8.81E-07
Generation of precursor metabolites and energy	1.55E-05
Purine-containing compound metabolic process	2.46E-05
Organic hydroxy compound metabolic process	2.66E-05
Sulfur compound metabolic process	3.08E-05
Cellular modified amino acid metabolic process	3.16E-05
Aspartate family amino acid metabolic process	5.21E-05
Cellular aldehyde metabolic process	4.28E-04
Monosaccharide metabolic process	4.61E-04
Response to extracellular stimulus	9.86E-04
Skin development	1.39E-03
Kidney development	2.26E-03
Multicellular organismal homeostasis	2.65E-03
Glutamine family amino acid metabolic process	4.35E-03
Inorganic cation import across plasma membrane	4.35E-03
Detoxification	4.51E-03
Organophosphate biosynthetic process	6.35E-03
Anion transport	9.66E-03

Lastly, there is an overabundance of nuclear genes locating to the mitochondria among dAD-enriched GO terms (cellular component, Supplementary Table S3). Though seemingly unsurprising given the mitochondrion’s role in metabolism, this includes mitochondrial rDNA genes (MRPS2, MRPS10; Table 1B) featuring extreme dAD despite little DE, suggesting allele-specific mitochondrial dysregulation, a field of recent interest [60]. Similarly, “neutrophil-mediated immunity” is found to be significantly enriched for dAD but not DE genes (Table 2). The role of this innate immune system component in cancer is unexplored yet emerging, as apparent from their relevance in cancer prognostics [61] and the novel oncological field of tumor-associated neutrophils [62]. Given the lack of relevant DE results here, further study of mitochondrial- and neutrophil-related genes in cancer may benefit from *maelstRom*’s allele-specific perspective.

## Discussion

The *maelstRom* software package introduced here is dedicated to the study of (differential) allelic dispersion. As this phenomenon is maintained through mitosis, dAD is indicative of early allele-specific aberrations but also RME (e.g., XCI). After benchmarking, we demonstrated that autosomal RME is rare at most in the kidney, with the clustered protocadherins as a likely exception. RME of the latter was already observed in Purkinje neurons, but additional experimental validation is required given their frequent (epi)genetic alterations in renal cancer. Of interest, long-range epigenetic silencing of these gene clusters has been observed in other cancer types, highlighting their relevance beyond neuronal tissue.

Among the top dAD genes in cancer, we found well-known causal cancer genes (FBP1, GSTP1), but also 2 of 3 components of the TP53-associated 3 M complex, and BSPRY, a poorly characterized gene that has repeatedly been associated with survival. Gene set analyses on all dAD genes revealed a strong metabolic focus (particularly the pentose phosphate pathway), contrasting DE’s propensity to return adaptive immunological genes. Combined, these results support a causal role for these top dAD genes during carcinogenesis. There results incentivize further research of poorly characterized top (BSPR, OPA1, ALPK3, etc.) but also less egregious dAD results.

### Methodological considerations

*maelstRom* is currently unique in its focus on AD as a gene-specific parameter of biological interest. Previous ASE studies largely ignored the concept of dispersion or only tested for deviation from the regular binomial distribution [63], which—as demonstrated in our results—is not even realistic for controls. Some ASE studies relied on the beta-binomial distribution but then simply incorporated AD as a constant nuisance parameter at either the SNP level [7, 64] or the gene level [65] (where it was used to combine per-SNP data to the gene level) rather than a biological interpretation. In this study, however, we demonstrated that AD can be used to study early allele-specific dysregulation, which makes them interesting candidates to be causally involved in these processes and thus targets for further study.

In its implementation, *maelstRom* strives for data efficiency and general applicability. Unlike other ASE modelers [7, 63], *maelstRom* requires no genotyping data, which not only implies a cost reduction and applicability on historic RNA-seq–only data but also returns results for loci not (sufficiently) covered by a genotyping assay. For example, in our study, 94% of analyzed SNPs were not part of TCGA’s supplied genotyping array data, which would otherwise be lost. Additionally, our *post hoc* SNP-to-gene combination [16] requires no allele-specific assemblies or complex preprocessing steps, while retaining a clear statistical interpretation. There are, however, some notable limitations to *maelstRom*’s current implementation as well. Genetically variable SNPs with more than 2 possible alleles cannot be modeled by 1 beta-binomial and require *maelstRom* to be fit in a pairwise fashion. Even more troublesome is too little genetic variation, as *maelstRom* cannot infer allele-specific parameters without heterozygous transcripts and thus cannot detect early (epi)genetic aberrations of their associated genes.

*maelstRom* is available as an R/C++ software package, providing fast, reliable results. Our SNP-to-gene combination hereto incorporates the score test (see Methods). C++ implementations (e.g., of the beta-binomial distribution) provide speed and numerical stability during optimization procedures. There is, to our knowledge, no existing modeling software that captures ASE as generically with minimal data (preprocessing) requirements.

### Biological considerations and perspectives

*maelstRom*’s strength is its ability to, as a population-level modeler, capture the variability of ASE (i.e., AD, in [diseased] populations). In accordance to our central crux (Fig. 1), this creates a “timeline of (epi)genetic dysregulation” throughout this population’s (or disease’s) development. However, this population variability approach means *maelstRom*’s output cannot be directly used as, for example, a per-individual clinical biomarker. It instead indicates candidate key genes in these phenomena’s general study. Of course, further scrutiny of such candidates may reveal biomarkers that are usable on a per-patient basis, such as direct detection of any of the (epi)genetic aberrations that ultimately influence *maelstRom*’s (d)AD results.

The aim of this publication was the introduction of *maelstRom*’s underlying rationales, its methodologies, and its results. We thus retained biological focus through the case study of a single phenomenon (AD increases due to epigenetic dysregulation) in a single cancer (KIRC). Pan-cancer application of *maelstRom* could, of course, deepen biological understanding of these results (e.g., which are kidney-specific, which are involved in general cancer). Similarly, we did not cover possible AD decreases. There are known cases of epigenetic regulation established very early in life being lost rather than gained in disease, such as loss of parental imprinting. This is, however, not in line with our case study’s main thesis (Fig. 1) and also, practically, rarely occurs (Fig. 6). In this limited setting, we understandably never gave hard cutoffs of what defines a “large,” “small,” or “regular” amount of (d)AD and mainly focused on its statistical significance. Though Fig. 6 contains several peaks of obviously “large” (d)AD, it also reveals control AD to be very different between the autosomal chromosomes and X chromosome (owing to the latter’s early-occurring XCI) and the interference of nonkidney cells on AD effect size (Fig. 6’s RME areas). This reveals a tissue’s developmental background, the timing of ASE occurrence, and tumor purity—thus also sampling strategy—can all influence AD, and thus we suggest its effect size (distribution) to be evaluated on a per-cohort basis.

The emerging single-cell transcriptomics seems greatly positioned to deal with at least the tumor purity issue—and it is, though this comes with some caveats. First is so-called allelic bursting, referring to single cells stochastically expressing genes in “bursts” of monoallelic transcription (or, at times, none at all). This complicates the study of true ASE in single-cell data and, though it has since been resolved, once led to abnormally high occurrences of RME being reported in single-cell studies [9, 66]. Recombination into pseudobulk can alleviate this issue but requires a sufficient amount of single cells per sample. Additionally considering *maelstRom*’s need for large enough populations to infer its parameters, we must conclude single-cell techniques and cohorts are currently very restrictive in both scope and financial cost for dAD studies, as in this article. Nevertheless, we acknowledge the technique’s potential for signal improvement and even tackling different research questions, for example, (d)AD across cells of a (developing) tissue through a combination with spatial transcriptomics.

Finally, care should be taken toward biologically “ranking” *maelstRom*’s results (in, e.g., order of importance). Besides the discussed effect size, dAD’s statistical significance is also affected by tumor purity, genotype frequency (number of heterozygotes), ASE type (e.g., copy number gain vs. loss), and the locus’s sequencing depth. Moreover, different genes of the same pathway may be dysregulated in different individuals across a population, a problem we addressed through gene set analysis. Lastly, dAD analysis remains a computational technique, which implies earliness of dysregulation. Though this makes its results interesting causal candidates in a studied phenomenon over, for example, reactive DE, earliness in no way equates causality, and further experimental validation remains indispensable in giving credence to any of its results.

## Conclusions

Generally, our results demonstrate that dAD should be considered side-to-side with DE when aiming to identify early aberrations in large-scale cancer transcriptomics studies. As dAD does not capture the impact of coding mutations, a strategy that also integrates mutation screening may identify oncogenic “double hits” of both alleles and yield a comprehensive overview of early dysregulation in cancer. Moreover, *maelstRom* is also applicable beyond oncology, since allele-specific dysregulation can equally occur during early development or in stem cells. Upon subsequent mitotic amplification, this results in so-called somatic mosaicism of allelic expression, which may contribute to disease or aging [67]. In conclusion, dAD analysis can provide an overview of early allele-specific dysregulation in development or disease, which may thus play a causal role in the processes, starting from solely population-scale bulk RNA-seq data. Given this broad relevance in transcriptomic studies, *maelstRom* is freely available from github.com/Biobix/maelstRom, and a step-by-step tutorial on its dAD analysis is hosted on biobix.github.io/maelstRom/articles/maelstRom_Allelic_Dispersion_tutorial.html.

## Methods

First, this Methods section provides thorough details regarding our Implementation section. As such, it is purposefully partly redundant with the latter regardinga general methodological description. While these details are not necessary to grasp *maelstRom*’s methodology and applicability, they do illustrate several important aspects our implementation takes into account but were not previously mentioned. We additionally report intermediary outcomes of several (sub)analyses when performed on our KIRC case study (see Results), for example, metaparameter estimates and the number of loci retained by filter criteria.

Second, we also provide methodological details that are not related to *maelstRom*’s new and unique implementation and analyses, but are nevertheless required to fully replicate the case study results, as presented in the main article. This includes the case study’s data acquisition and preprocessing, as well as standard, well-established (not *maelstRom*-specific) analyses thereon (e.g., differential expression analysis, gene-level promoter hypermethylation analysis). This concludes with a table documenting used (and created) data sets, software, and websites in, or by, our publication.

*maelstRom*’s original source code is available at GitHub. It is an R software package, with several subroutines implemented in C++ for increased computational speed and precision during numerical optimization (during which extreme parameter values can be encountered and subsequently results in catastrophic cancellation or boundary issues if not properly accounted for). A tutorial of *maelstRom*’s AD-analyzing functionalities on a toy dataset is available at GitHub.

### Implementation details

#### Metaparameter estimation

Reconsider *maelstRom*’s core ASE model from the Implementation section (Equation M1):


(M1) \begin{eqnarray*}
PMF\left({x}_r,{x}_v \right) &=& {\phi }_{rr}*{BetaBin}\left({x}_r|n = {x}_r + {x}_v,\,\, \pi = 1 - SE,\,\, \rho = {\rho }_{hom} \right)\\
&& + {\phi }_{rv}*{BetaBin}\left({x}_r|n = {x}_r + {x}_v,\,\, \pi = {\pi }_{het} = {\boldsymbol AB},\right.\\
&& \left.\qquad\qquad\qquad \rho = {\rho }_{het} = {\boldsymbol AD} \right)\\
&& + {\phi }_{vv}*{BetaBin}\left({x}_r|n = {x}_r + {x}_v,\,\, \pi = SE,\,\, \rho = {\rho }_{hom} \right)\\
\end{eqnarray*}

This model relies on 1 metaparameter (constant across loci): sequencing error (*SE*), which conceptually corresponds to its namesake (probability of variant SNP counts being reported in reference SNP homozygotes or vice versa), though it captures other technical errors too (e.g., alignment errors). While this metaparameter can be fixed based on expert knowledge or sequencing technology specifications, we generally recommend its empirical estimation, as well as not setting it extremely low (not below 0.002) to allow flexibility in sample assignment during AD analyses by expectation–maximization (EM).

To estimate SE, *maelstRom* fits a simplified Equation M1 to all loci using EM (see Implementation section for an explanation of all parameters):


(M2) \begin{eqnarray*}
PMF\left({x}_r,{x}_v \right) &=& {\phi }_{rr}*{Binomial}\left({x}_r|n = {x}_r + {x}_v,\,\, p = 1 - SE \right)\\
&& + {\phi }_{rv}*{Binomial}\left({x}_r|n = {x}_r + {x}_v,\ p = 0.5 \right)\\
&& + {\phi }_{vv}*{Binomial}\left({x}_r|n = {x}_r + {x}_v,\ p = SE \right)
\end{eqnarray*}

This differs from Equation M1 by assuming perfectly balanced allelic expression (p-parameter of 0.5) in heterozygotes and disregarding nonrandom sampling variance by using regular binomials. The latter effectively means that every (biologically) distinct sample is treated as a technical replicate of 1 (biologically) identical sample. Even in the absence of ASE effects increasing allelic dispersion, such an assumption is overly simplistic and will be violated by many loci (see Results). However, fitting Equation M2 is very fast compared to Equation M1 due to the existence of analytical solutions for binomial parameter maximum likelihood estimates. Also, *SE* is a homozygote-specific parameter, whose fit is less affected by these violations. Thus, by retaining only high-quality loci with no egregiously unrealistic SE estimate (estimated *SE* < 0.035, locus must be covered by over 40 samples, median per-sample coverage $\ge $10, minor allele count fraction over all samples $\ge $0.15), we obtain a robust median *SE* estimate. Simultaneously, we estimate the inbreeding coefficient on these same filtered loci, using Equation M2’s mixture weights (${\phi }_{rr}$, ${\phi }_{rv}$, ${\phi }_{vv}$). This is to be used in future filter criteria:


(M3) \begin{eqnarray*}
\begin{array}{@{}*{1}{c}@{}} {{F}_{\textit{inbr}} = 1 - \frac{{\textit{observed}\ \textit{heterozygosity}}}{{\textit{expected}\ \textit{hetezozygosity}\ \textit{assuming}\ \textit{panmixis}}}}\\ { = 1 - \frac{{{\phi }_{rv}}}{{2*\left( {{\phi }_{rr} + {\phi }_{rv}/2} \right)*\left( {{\phi }_{vv} + {\phi }_{rv}/2} \right)}}} \end{array}
\end{eqnarray*}

The population’s inbreeding coefficient (${F}_{\textit{inbr}}$) is obtained as the median of these per-locus estimates. For the “random” human population in our KIRC study, panmixis would be a valid prior assumption (${F}_{\textit{inbr}} = 0$). Nevertheless, *maelstRom* provides this estimator for application on other (artificial) populations or simply as a sanity check. In our KIRC case study data, this procedure yields *SE* and ${F}_{\textit{inbr}}$ estimates of 0.00220 and 0.0104, respectively.

#### Differential allelic dispersion detection

Reconsider *maelstRom*’s *differential* AD model, which is fit to per-locus, per-sample allele counts using the EM algorithm (Equation M4):


(M4) \begin{eqnarray*}
PMF\left({x}_r,{x}_v \right) &=& {\phi }_{rr}*{BetaBin}\left({x}_r|n = {x}_r + {x}_v,\,\, \pi = 1 - SE,\,\, \rho = {\rho }_{hom} \right)\\
&& + {\phi }_{rv}*{BetaBin}\left({x}_r|n = {x}_r + {x}_v,\,\, \pi = {\pi }_{het},\right.\\
&&\left.\qquad\qquad\qquad \rho = {I}_{control}{\rho }_{het,control} + {I}_{case}{\rho }_{het,case} \right)\\
&& + {\phi }_{vv}*{BetaBin}\left({x}_r|n = {x}_r + {x}_v,\,\, \pi = SE,\,\, \rho = {\rho }_{hom} \right)\\
\end{eqnarray*}

Here, ${I}_{\textit{control}}$ and ${I}_{\textit{case}}$ are indicator variables designating the subpopulation a sample belongs to. Aspects of special interest regarding this model fit are discussed below, though *maelstRom*’s full algorithm can be downloaded from GitHub.

1. Several beta-binomial probability mass function (PMF) implementations are available for R, yet proved insufficient for *maelstRom* because of their slow computation speed and/or returning nonsensical results for extreme parameter values (e.g., *VGAM*’s implementation [68] defaults to the binomial PMF in such cases). While these extreme parameters are usually not realistic, they can be encountered during numerical optimization’s exploration of the parameter space (especially when optimizing log- and logit-transformed parameters), in which case faulty PMF and PMF gradient values can lead to errors, getting stuck in unoptimized parameters, or exploring the parameter space in the wrong (nonoptimizing) direction. As such, *maelstRom* implements its own beta-binomial PMF as either a long product [69]:
  (M5) \begin{eqnarray*}
PM{F}_{\textit{BetaBin}}\left( {x;n,\pi ,\theta } \right) = \ \left( {\begin{array}{@{}*{1}{c}@{}} n\\ x \end{array}} \right)\frac{{\mathop \prod \nolimits_{k = 0}^{x - 1} \left( {\pi + k\theta } \right)\mathop \prod \nolimits_{k = 0}^{n - x - 1} \left( {1 - \pi + k\theta } \right)}}{{\mathop \prod \nolimits_{k = 1}^{n - 1} \left( {1 + k\theta } \right)}}\\
\end{eqnarray*}
  or via beta-functions:
  (M6) \begin{eqnarray*}
\ PM{F}_{\textit{BetaBin}}\left( {x;n,\pi ,\theta } \right) = \ \frac{n}{{x*\left( {n - x} \right)}}\frac{{\textit{beta}\left( {1/\theta ,\ n} \right)}}{{\textit{beta}\left( {\pi /\theta ,x} \right)*\textit{beta}\left( {\left( {1 - \pi } \right)/\theta ,n - x} \right)}}\\
\end{eqnarray*}

$\theta $ and $\rho $ (the latter being the overdispersion parameter used until now) are straightforward transformations of one another: both model AD, but $\theta $ ranges from 0 to infinity, and $\rho $ ranges from 0 to 1 (specifically, $\rho = \theta /( {1 + \theta } )$). Using $\theta $ often results in much simpler mathematical expressions and thus is preferred throughout the Methods and in *maelstRom*’s implementation.
  Equation M5 gives an exact beta-binomial PMF value but is slow to calculate and thus is only used for beta-binomial PMF values (and its likelihood derivatives, used in, e.g., optimization algorithms and statistical tests) in case of parameter values for which Equation M6 cannot be calculated. Otherwise, *maelstRom* uses Equation M6, which is approximate in its implementation as beta-function values are, themselves, calculated numerically in R. All PMF calculations are $log$-transformed to accommodate extreme values, but overly extreme values can still lead to numerical errors by catastrophic cancellation of $logbeta$ terms (Equation M6). *maelstRom* addresses this issue, in beta-binomial PMFs and our implementations of their gradients, by (1) assessing whether catastrophic cancellation occurs at the default double precision of 64 bits; if so, (2) assessing whether Taylor polynomial approximations of the PMF (gradient) decay sufficiently rapid for a third-order approximation’s error to be negligible; and if not, (3) resort to the C++ boost multiprecision library [70] to increase the $logbeta$ terms’ numerical precision. This is a last resort, as the computation time scales with the number of bits used. Even so, *maelstRom* places a limit on the bits used per number (2,048) so as to not overload RAM, defaulting to the regular binomial PMF if this is exceeded, though such a scenario is highly improbable in practice.
2. Initial estimates for $\pi $- and $\theta $-parameters (which is necessary as an input to numerical optimization algorithms) are obtained via moment estimators; this is nontrivial for beta-binomial samples with differing *n* (total allele count), but based on Kleinman’s work [11], we derived (all big summations are over all *S* samples):
  (M7) \begin{eqnarray*}
\hat{\pi } = \frac{{\sum {w}_s\left( {{{\hat{p}}}_s} \right)}}{{\sum {w}_s}}
\end{eqnarray*}
   
  (M8) \begin{eqnarray*}
\hat{\theta } = \ 1/\left( {\frac{{\hat{\pi }*\left( {1 - \hat{\pi }} \right)*\left( {\sum {w}_s{z}_s - \sum {w}_s{z}_s/{n}_s} \right)}}{{Q - \ \hat{\pi }*\left( {1 - \hat{\pi }} \right)*\left( {\sum {w}_s{z}_s/{n}_s} \right)}} - 1\ } \right)
\end{eqnarray*}
  where ${\hat{p}}_s = ( {{x}_{s,ref}/{n}_s} )$ is the fraction of reference reads in sample *s*; $Q = \ \sum {w}_s{( {{{\hat{p}}}_s - \hat{\pi }} )}^2$ is a weighted sum-of-squares, ${z}_s = \ ( {1 - {w}_s/\sum {w}_s} )$; and ${w}_s$ is a per-sample weight between 1 and ${n}_s$ for which Kleinman suggests an iterative procedure. However, given our aim to find a rough initial estimate, all ${w}_s$ are simply set to 1, which corresponds to Kleinman’s ideal weights for a $\theta = 0$ scenario.
3. *maelstRom* uses the Broyden–Fletcher–Goldfarb–Shanno (BFGS) algorithm for numerical optimization during EM interations (GNU Scientific Library’s [71] bfgs2 implementation). Though $\pi $ and $\theta $ are, respectively, bounded and left-bounded ($\pi \in[ {0,1} ];\ \theta \in[ {0, + \infty } [$), we avoided bounded optimization algorithms as they proved either slow or numerically unreliable, opting for parameter transformation ($logit( \pi )$; $log( \theta )$) prior to numerical optimization, instead.
4. Beta-binomial PMFs are bimodal when $\theta > {\mathrm{max}}( {\pi ,1 - \pi } )$. Such bimodality of the heterozygous peak is undesirable in *maelstRom*. One could argue this might enable *maelstRom* to also model complete allele-specific imprinting in heterozygotes; yet, in practice, allowing bimodality mainly results in the optimization algorithm using the heterozygous mixture component to fit homozygous data during early iterations, then getting stuck in this wrong (local) parameter optimum. As such, *maelstRom* reruns EM using *alabama*’s [72] (R package) augmented Lagrangian algorithm if bimodality occurs. This allows for nonlinear constraints in parameters ($\theta \le max( {\pi ,1 - \pi } )$) but is considerably slower than GSL’s bfgs2 and thus not the default choice.
5. *maelstRom* uses a robust EM implementation, which excludes extremely influential observations based on sample deletion estimates (Cook [12]). In short, after estimating parameters on the full dataset (${\hat{\pi }}_{het}$, ${\hat{\theta }}_{het}$), they are reestimated on data subsets that, one-by-one, leave out every sample individually (${\hat{\pi }}_{het,j}$, ${\hat{\theta }}_{het,j}$ when leaving out sample *j*). The differences between these estimates (${\hat{\pi }}_{het} - {\hat{\pi }}_{het,j}\ $ and ${\hat{\theta }}_{het} - {\hat{\theta }}_{het,j}$) reflect sample *j*’s leverage on parameter estimation, with *maelstRom* deeming the sample an outlier if any parameter’s difference is greater than 5 times the sample standard deviation of all its differences across samples.

This outlier detection happens separately on the control and case data, given their ASE parameters can be very different due to dAD. Then, nonoutlying samples are recombined to perform EM fits on the following: once with a shared AD parameter (Equation M1) and once with a separate one for controls and cases (Equation M4). A standard likelihood ratio test on these 2 fits can test the current locus for dAD (1 degree of freedom). An important note here is that the LRT is unreliable when testing near-boundary parameter values, such as $\theta = 0$, yet such absence of biological variability is not realistic in our setting of heterogeneous populations. Nevertheless, we caution against using this test against such “absence of biological variability $\theta = 0$” hypotheses.

Outlying samples are marked as such, so they can be excluded from any further analyses but are visible in *maelstRom*’s final output for accurate assessment of HWE (see below: as ${\pi }_{het}$ and ${\theta }_{het}$ are parameters of the heterozygous mixture component, outlier detection mainly affects heterozygotes, which would bias HWE assessment when simply removed). Note that *maelstRom*’s outlier detection requires many per-sample refits per locus and thus has a significant computational cost; end users may opt for simpler outlier detection (e.g., simply based on total allele count) or forego it entirely.

#### SNP-to-gene combination

As discussed in the Implementation section, combining *maelstRom*’s per-SNP *P* values is not trivial. A (weighted) mean *P* value cannot consolidate independent statistical evidence for dAD across SNPs into a stronger conclusion, as a mean can never be lower than the minimum of the *P* values being combined. Other standard *P* value combinations such as Fisher’s method (and its weighted variant, the Lancaster method) assume complete independence among combined tests, which is not suitable for (proximal) SNPs whose allele counts can originate from the same RNA molecule or even the same sequencing read. To accommodate for this dependence among SNPs, Dai et al. [16] provide correlated Lancaster methods.

The Lancaster test statistic is defined as


(M9) \begin{eqnarray*}
{T}_{Lan} = \mathop\sum\nolimits_{i = 1}^N \gamma _{\left( {{w}_i/2,\ 2} \right)}^{ - 1}\left( {1 - {p}_i} \right)
\end{eqnarray*}

with *N* the number of tests being combined, ${p}_i$ the *P* value corresponding to the *i*th test, and $\gamma _{( {{w}_i/2,\ 2} )}^{ - 1}$ the inverse gamma distribution’s cumulative distribution function (CDF) with a shape parameter of ${w}_i/2$ (${w}_i$ being the weight assigned to the *i*th *P* value) and a scale parameter of 2. When all *N* tests are independent, and their (shared) null hypothesis is true, ${T}_{Lan}$ has a chi-square distribution with $\mathop \sum \limits_{i = 1}^N ( {{w}_i} )$ degrees of freedom. When there is dependence among tests, it does not, and Dai et al. [16] propose a modified statistic with an approximate distribution:


(M10) \begin{eqnarray*}
{T}_{\textit{ModLan}} = c*{T}_{Lan} \approx \chi _v^2\ \textit{when}\ {H}_0\ is\ \textit{TRUE}
\end{eqnarray*}

with $\chi _v^2$ a chi-square distribution with *v* degrees of freedom, and


(M11) \begin{eqnarray*}
c &=& \frac{v}{{E\left[ {{T}_{Lan}} \right]}}\\
v &=& 2*\frac{{{{\left( {E\left[ {{T}_{Lan}} \right]} \right)}}^2}}{{var\left( {{T}_{Lan}} \right)}}\\
E\left[ {{T}_{Lan}} \right] &=& \mathop \sum \nolimits_{i = 1}^N \left( {{w}_i} \right)\\
var\left( {{T}_{Lan}} \right) &=& 2*\mathop \sum \nolimits_{i = 1}^N \left( {{w}_i} \right) + 2*\mathop \sum \nolimits_{i < j} \textit{Covar}_{i,j}\\
{Covar}_{i,j} &=& cov\left( {\gamma _{\left( {{w}_i/2,\ 2} \right)}^{ - 1}\left( {1 - {p}_i} \right),\ \gamma _{\left( {{w}_j/2,\ 2} \right)}^{ - 1}\left( {1 - {p}_j} \right)} \right)
\end{eqnarray*}

Dai et al. [16] provide (references to) derivations of these expressions; $\mathop \sum \limits_{i < j} \textit{Covar}_{i,j}$ corresponds to the summed off-diagonal elements of the variance–covariance matrix (i.e., only the covariances) of gamma-transformed per-SNP *P* values. These covariances are used to adjust the combined *P* value for dependency among SNPs. In an ideal world, these are known: given in-depth expert knowledge of the distance between 2 SNPs, how this impacts the correlation of their RNA-seq allelic reads through the transcription process, and how this ultimately impacts the correlation of dAD testing *P* values obtained from likelihood ratio tests of beta-binomial mixture models fit to said allelic reads, this may be theoretically possible. In practice, though, it is more realistic to empirically estimate $\mathop \sum \limits_{i < j} \textit{Covar}_{i,j}$ through Dai et al.’s [16] proposed permutation procedure.

For a certain gene of interest, each permutation iteration sees sample labels (i.e., which samples are considered controls and which are considered cases) randomly reaassigned, but this sample reassignment is shared across SNPs within 1 iteration. This effectively breaks any data correlation patterns due to real, biological differences dictated by the shuffled label (i.e., dAD due to biology) but retains correlation patterns due to inter-SNP dependency. Redoing *maelstRom*’s entire dAD analysis on this permutated data is thus expected to produce nonsignificant *P* values (more exactly: *P* values that are uniformly distributed between 0 and 1), but said *P* values will be similar for correlated SNPs within 1 permutation. Thus, after many permutations (10,000 for this publication), $\mathop \sum \limits_{i < j} \textit{Covar}_{i,j}$ can be estimated from the *P* values’ empirical covariance matrix.

Considering the substantial computational cost of *maelstRom*’s numerical procedures, repeating the dAD fit 10,000 times for every gene in full-genome data, just to adjust results for inter-SNP dependency, is impractical. As such, we expanded Dai et al.’s [16] permutation procedure by using the score test, instead of the LRT, when calculating each permutation’s *P* values. While asymptotically equivalent, these tests differ in their required fits: the LRT requires optimized model fits under both the null and alternative hypotheses (cf. Equations M1 and M4: ${\rho }_{het,\ \textit{control}} = {\rho }_{het,\ \textit{case}}$, respectively, ${\rho }_{het,\ \textit{control}}\not={\rho }_{het,\ \textit{case}}$); the score test relies on the null hypothesis fit alone. Though this makes the score test redundant for most applications (the alternative model is usually the one of actual interest, e.g., the occurrence of dAD and its estimated $\rho $ values in cases and control), it is useful here: the null hypothesis model fit is identical for all sample label permutations as it assumes ${\rho }_{het,\ \textit{control}} = {\rho }_{het,\ \textit{case}}$ (and all other parameters are already not label-specific), so it needs to be fit only once. At the same time, for the purpose of dependence correction, we are not interested in optimized parameter values (and therefore the alternative model fit) of every permutation, only in *P* values. Relying on the aforementioned asymptotic equality of both tests, we argue that the use of score test–derived permutation *P* values to adjust our LRT-derived results is also asymptotically valid. Its implementation specifics are provided below.

For any SNP, given reference and variant allelic counts $\mathop x\limits^{\rightarrow} = ( {{x}_1 = \{ {{x}_{r,1};{x}_{v,1}} \},{x}_2,\ldots,{x}_S} )$ across all *S* samples, as well as the vector of parameter values for *maelstRom*’s Equation M4  $\mathop \tau \limits^{\rightarrow} $ (containing actually fitted parameters: ${\pi }_{het}$, ${\rho }_{hom}$, ${\rho }_{het,\textit{control}}$, ${\rho }_{het,\textit{case}}$, ${\phi }_{rr}$, and ${\phi }_{rv}$; the last mixture component, ${\phi }_{vv}$, is entirely dependent on the other two: ${\phi }_{vv} = 1 - {\phi }_{rr} - {\phi }_{rv}$), we define $P( {{x}_s,\mathop \tau \limits^{\rightarrow} } )$ as the probability of the allelic count in sample *s* (i.e., its Equation M4 PMF value). From this, the log-likelihood of observation $\mathop x\limits^{\rightarrow} $ equals:


(M12) \begin{eqnarray*}
l\left( {\mathop x\limits^{\rightarrow} ,\ \mathop \tau \limits^{\rightarrow} } \right) = \mathop \sum \nolimits_{s = 1}^S log\left( {P\left( {{x}_s,\mathop \tau \limits^{\rightarrow} } \right)} \right)
\end{eqnarray*}

The efficient score of the *t*th parameter in $\mathop \tau \limits^{\rightarrow} $ (${\tau }_t$) is


(M13) \begin{eqnarray*}
{v}_t\left( {\mathop x\limits^{\rightarrow} ,\mathop \tau \limits^{\rightarrow} } \right) = \frac{1}{{\sqrt S }}\frac{{\delta l\left( {\mathop x\limits^{\rightarrow} ,\ \mathop \tau \limits^{\rightarrow} } \right)}}{{\delta {\tau }_t}} = \frac{1}{{\sqrt S }}\left( {\mathop \sum \nolimits_{s = 1}^S \frac{1}{{P\left( {{x}_s,\mathop \tau \limits^{\rightarrow} } \right)}}\frac{{\delta P\left( {{x}_s,\mathop \tau \limits^{\rightarrow} } \right)}}{{\delta {\tau }_t}}} \right)\\
\end{eqnarray*}

with $\mathop V\limits^{\rightarrow} $ the vector of efficient scores of all *T* parameters ($T = 6$ for Equation M4):


(M14) \begin{eqnarray*}
\mathop V\limits^{\rightarrow} \left( {\mathop x\limits^{\rightarrow} ,\mathop \tau \limits^{\rightarrow} } \right) = \left( {{v}_1\left( {\mathop x\limits^{\rightarrow} ,\mathop \tau \limits^{\rightarrow} } \right),\ldots,{v}_T\left( {\mathop x\limits^{\rightarrow} ,\mathop \tau \limits^{\rightarrow} } \right)} \right)
\end{eqnarray*}

The Fisher information matrix *I*’s *r*th row, *k*th column element equals (if $log( {P( {X,\mathop \tau \limits^{\rightarrow} } )} )$ is twice differentiable to all parameters in $\mathop \tau \limits^{\rightarrow} $ and under certain regularity conditions [73]):


(M15) \begin{eqnarray*}
I{\left( {\mathop \tau \limits^{\rightarrow} } \right)}_{r,s} = - E\left[ {\frac{{{\delta }^2}}{{\delta {\tau }_r\delta {\tau }_s}}log\left( {P\left( {X,\mathop \tau \limits^{\rightarrow} } \right)} \right)} \right]
\end{eqnarray*}

This expected value should be calculated at the supposed “true” value of $\mathop \tau \limits^{\rightarrow} $ (though a consistent estimator of the latter is sufficient for maintaining the asymptotic properties of whatever statistic *I* is used in), and under the assumption, *X* is exactly $P( {X,\mathop \tau \limits^{\rightarrow} } )$ distributed.

Using these definitions, and given (maximum likelihood) estimates of $\mathop \tau \limits^{\rightarrow} $ under both the null (${\mathop \tau \limits^{\rightarrow} }_{H0};$ imposing ${\rho }_{het,\ \textit{control}} = {\rho }_{het,\ \textit{case}}$) and alternative hypotheses (${\mathop \tau \limits^{\rightarrow} }_{H1};$  ${\rho }_{het,\ \textit{control}}\not={\rho }_{het,\ \textit{case}}$), the LRT statistic is obtained as


(M16) \begin{eqnarray*}
{\lambda }_{LRT} = 2*\left( {l\left( {\mathop x\limits^{\rightarrow} ,{{\mathop \tau \limits^{\rightarrow} }}_{H1}} \right) - l\left( {\mathop x\limits^{\rightarrow} ,{{\mathop \tau \limits^{\rightarrow} }}_{H0}} \right)} \right)
\end{eqnarray*}

The score test statistic is obatined as


(M17) \begin{eqnarray*}
{\lambda }_{ST} = \mathop V\limits^{\rightarrow} {\left( {\mathop x\limits^{\rightarrow} ,{{\mathop \tau \limits^{\rightarrow} }}_{H0}} \right)}^{\prime} \cdot I{\left( {{{\mathop \tau \limits^{\rightarrow} }}_{H0}} \right)}^{ - 1} \cdot \mathop V\limits^{\rightarrow} \left( {\mathop x\limits^{\rightarrow} ,{{\mathop \tau \limits^{\rightarrow} }}_{H0}} \right)
\end{eqnarray*}

with both being asymptotically ${\chi }^2$ distributed under the null hypothesis with degrees of freedom equal to the number of restrictions imposed by said null hypothesis (here 1; ${\rho }_{het,\ \textit{control}} = {\rho }_{het,\ \textit{case}}$), from which *P* values can be derived.

In practice, *maelstRom* uses the negative Hessian instead of Fisher information when calculating the score test statistic (Equation M17), as the Fisher information’s (algebraic or numeric) computation is rather bothersome. This does not affect the asymptotic properties of the score test [74]. The Hessian’s value in its *r*th row, *k*th column is


(M18) \begin{eqnarray*}
H{\left( {\mathop x\limits^{\rightarrow} ,\mathop \tau \limits^{\rightarrow} } \right)}_{r,s} = \frac{{{\delta }^2l\left( {\mathop x\limits^{\rightarrow} ,\ \mathop \tau \limits^{\rightarrow} } \right)}}{{\delta {\tau }_r\delta {\tau }_s}}
\end{eqnarray*}

This is, finally, how *maelstRom* obtains *P* values for inter-SNP dependence correction, relying solely on the null hypothesis fit, which is the same in every permutation. Not that this does, of course, not mean that score test statistics (Equation M17) are identical across permutations: even though the null hypothesis imposes ${\rho }_{het,\ \textit{control}} = {\rho }_{het,\ \textit{case}}$, these $\rho $s are still considered separate parameters in Equation M17’s first- and second-order derivatives; thus, these derivatives are affected by the sample label permutation and need to be recalculated. It is only the EM-optimized null hypothesis parameters (${\mathop \tau \limits^{\rightarrow} }_{H0}$) that remain constant, but this EM fit is the most computationally intensive step anyway; Equation M17’s recalculation is not.

Some final, important implementation details of *maelstRom*’s SNP-to-gene combination are as follows:

1. Before SNP-to-gene combinatio n, additional filter criteria are imposed on the remaining 127,023 autosomal and 2,083 X chromosomal SNPs. (1) A sample median total allelic count $\ge $4 in both cases and controls; (2) an estimated number of heterozygotes in both cases and controls $\ge $12 for autosomal genes and $\ge $8 for X chromosomal genes (the latter being less strict due to only 20 female control samples being available); (3) having a final fitted $0.05 \le {\pi }_{het} \le 0.95$ (Equation M4), as a more extreme ${\pi }_{het}$ likely indicates a failed model fit (the heterozygous mixture component being used to fit homozygous data) or otherwise complete absence of heterozygous individuals; and (4) both control and case data adhering to HWE, defined as having a *P* value $> $0.001 for a chi-square frequency table test comparing EM-fitted genotype frequencies (performed on control and case data separately, which already happened for outlier detection) to those expected under HWE with a population inbreeding coefficient, as determined during metaparameter estimation (Equation M3). This is a statistically weak conclusion (accepting the null hypothesis of HWE) but a standard filter for HWE conformity nevertheless [75]. These filters retain 60,089 and 1,015 autosomal and X chromosomal SNPs, respectively. Outlying samples are considered when testing for HWE but are not considered in any other filters or calculations in *maelstRom*’s entire pipeline.
2. SNPs are assigned to genes based on their NCBI dbSNP annotation [19]; if a SNP is listed as a genetic variant of multiple genes, it is assigned to the gene for which it most likely occurs in (processed) mRNA, based on a hierarchy of {exonic variants} > {3′ and 5′ UTR and noncoding transcript variants} > {intronic, splice donor, and splice acceptor variants} > {long-distance up- and downstream variants}. If dbSNP does not provide gene annotation, chromosome position-based annotation using R’s *GenomicRanges* [76] is attempted (prioritizing “exonic” over “nonexonic” annotations). Hereafter, nonannotated SNPs and SNPs mapping to multiple genes (at the same aforementioned hierarchical level) are filtered out, which leaves 55,773 autosomal SNPs corresponding to 12,079 unique genes and 967 X chromosomal SNPs corresponding to 311 genes.
3. As Lancaster weights (${w}_i$; Equation M9) for SNPs of a gene, we use the control or case (whichever is lower, as this one is “limiting” to the reliability of the SNP’s result) estimated number of heterozygotes times median allelic count. These SNP weights are then, per-gene, rescaled to sum to 2 times the amount of SNPs annotated to the gene, in accordance to the ideal weighting scheme proposed by Yoon et al. [77] (this scheme makes it so the Lancaster method is equivalent to the Fisher method in the case of equally weighted SNPs, which it, reasonably, should be).
4. While iterative sample label permutations are done randomly, a permutation is redone if the reassigned control or case group contains 6 or fewer putative heterozygotes (according to the separate control and case beta-binomial mixture fits performed during outlier detection), which would make a differential test on Equation M4’s heterozygous parameters difficult. Similarly, a permutation is redone if it results in a (numerically) noninvertible Hessian, which makes computation of the score test statistic (Equation M17) impossible.
5. If any $Cova{r}_{i,j}$ (Equation M11) is calculated to be negative, this is deemed biologically unrealistic and set to zero (if a certain gene is transcribed in a certain way or rate in a given sample, we expect this sample’s data across SNPs to be similar and thus positively correlated or, at the very least, not negatively correlated).
6. Per-gene results other than *P* values are obtained as weighted arithmetic means of the corresponding per-SNP results. This includes, in controls, the estimated number of heterozygotes, median total allelic count, and ${\rho }_{het}$ estimates, in which each SNP’s contribution is weighted by the square root of its estimated number of heterozygotes times median total allelic count in controls. The same measures are also combined for cases, using analogous per-SNP weights, but calculated on case data.
7. It is only after SNP-to-gene combination that gene-level dAD-detecting *P* values are FDR-corrected using the Benjamini–Hochberg procedure over all 12,079 autosomal genes (though our final SNP-level results also contain dAD-detecting *P* values that are, themselves, FDR-corrected at the said SNP level). To offset the inherently low-powered dAD detection for the X chromosome (having only 20 female control samples, implying even less control heterozygotes), as well as considering they are analyses on effectively different datasets and the fact that the X chromosome is entirely disregarded in all biological interpretations of dAD results in any remaining analyses (selecting top dAD hits, gene set overrepresentation analysis), FDR correction was performed on the 311 X chromosomal genes separately. dAD-testing *P* values listed in the main text are these gene-level FDR-corrected *P* values.

*maelstRom*’s final results are, however, reported on only 11,325 autosomal and 291 X chromosome genes (instead of the 12,079 and 311 just mentioned). This is because DE analyses (which were cross-referenced with dAD results) use a Xenabrowser-provided htseq gene count file (see Data and Analysis Software Details section), which provided gene counts for only these 11,325 of 12,079 autosomal genes and 291 of 311 X chromosome genes.

#### Canonical dAD

“Canonical” dAD is described in the main text as the correlation between a sample’s contribution to (increased) AD and its expression. For a certain SNP, the latter simply equals each of its samples’ total allelic counts. The former is calculated as the inverse of a sample’s minimal tail area according to final fit of the heterozygous mixture component in Equation M4 (the inverse, so that a greater value corresponds to greater extremity and thus a greater contribution to increased AD). In other words: (the inverse of) the minimum of Equations M4’s heterozygous mixture component’s cumulative mass function (CMF) and 1 minus this same CMF value plus the heterozygous mixture component’s PMF value in this sample (its complement, Fig. 4; adding the final term is necessary for a fair comparison between both tail values, as the PMF in the sample itself is, by default, included in the computation of the lower tail but not in the upper tail area for discrete distributions). Only case samples are considered when assessing canonical dAD.

All correlations and corresponding *P* values were calculated with R’s *cor.test* function using Spearman rank correlation. Only heterozygous samples (higher chance to be heterozygous than either homozygote based on Equation M4’s fit) and having a total allelic count $> $20 are considered; if an SNP has no such samples, its correlation and *P* value (${\mathrm{Cor}}{{\mathrm{r}}}_{{\mathrm{canon}}}$ and ${{\mathrm{p}}}_{{\mathrm{canon}}}$ in Table 1, main text) are reported as *NA*. For every gene, only the most significant correlation across SNPs is reported, but FWER-corrected (Holm’s method) across SNPs within a gene.

“Canonical” dAD was combined with other filter criteria to retain top dAD results from the final (filtered) table of 11,325 autosomal genes (Supplementary Data 1). To avoid causally irrelevant false positives, the most prevalent CNA regions in stage 1 KIRC were excluded, visually guided by Figs. 5 and 6: chromosome 3p and the first part of 3q ($\le $130 M bp), 5q ($> $51.4 M bp), 6q ($> 100\ {\mathrm{M\ }}$ bp), 8p ($\le \ 45.2\ {\mathrm{M\ }}$ bp), 9p ($\le \ 43\ {\mathrm{M\ }}$ bp), and 14q ($> $ 17.2 M bp). Top genes have dAD results based on at least 2 SNPs, have a median sample allele count $\ge $10 and expected number of heterozygotes $\ge $15 in both controls and cases, ${\rho }_{het,\ \textit{case}} \ge 1.5{\rho }_{het,\ \textit{control}}$, ${\rho }_{het,\ \textit{case}} \ge 0.05$, and a dAD-detecting (FDR-corrected) *P* value $< $1E-10. Finally, genes in Table 1A (Results) have at least 1 SNP with a statistically significant ${{\mathrm{p}}}_{{\mathrm{canon}}}$ at the 5% FWER level (Holm’s method across SNPs per gene), with ${\mathrm{Cor}}{{\mathrm{r}}}_{{\mathrm{canon}}}$ acting in the same direction as global DE ($sign( {{\mathrm{Cor}}{{\mathrm{r}}}_{{\mathrm{canon}}}} ) = \ \textit{sign}( {lo{g}_2( {FC} )} )$). Note that multiple testing correction across all SNPs or genes present in the dataset would be too strict for this exploratory test, which is inherently low-powered due to needing sufficient heterozygous samples per SNP (rather than allele counts, on top of number of samples, for dAD tests) and confounding factors (reactive DE, tumor purity).

#### Gene Ontology overrepresentation analyses

GO ORAs were performed on the final (filtered) table of 11,325 autosomal genes, considering 3 gene sets: one featuring significant DE (2,207 genes, ${p}_{DE,\ FDR}$  $< $ 0.001 and $abs( {lo{g}_2( {FC} )} ) > 1$), one featuring significant dAD (2,142 genes, ${p}_{dAD,FDR} < $ 0.001 and ${\rho }_{het,\ \textit{case}} \ge 1.5{\rho }_{het,\ \textit{control}}$), and one featuring both (942 genes, ${p}_{DE,FDR}$  $< $ 0.05 and $abs( {lo{g}_2( {FC} )} ) > $ 0.5 and ${p}_{dAD,FDR}$  $< $ 0.001 and ${\rho }_{het,\ \textit{case}} \ge 1.5{\rho }_{het,\ \textit{control}}$, with less stringent DE significance criteria for the latter to retain sufficient genes). For GO ORA, the *enrichGO* function of R’s *clusterProfiler* package [78] was used with default settings and using the full set of 11,325 autosomal genes as background. To reduce redundancy among enriched GO terms, we removed any terms for which $> $60% of genes contributing to said term’s enrichment were present in a more significantly enriched GO term.

#### Allele fraction plots

Allele fraction plots (Fig. 7, results) depict the observed distribution of allele fractions as a histogram, but also *maelstRom*’s model fit as lines. Besides the beta-binomial distribution being a discrete distribution, its shape also depends on the underlying total count *n*, which varies across samples. For these visualizations, we used the median total allele count (determined separately in cases and controls) of the SNP being plotted when visualizing the fitted distributions as line plots.

### Data and analysis software details

#### Data acquisition

TCGA RNA-seq BAM files (aligned to GRCh38) and the associated GRCh38 Reference Sequence were downloaded from the GDC data portal [17]. The former consist of 72 control KIRC samples and 268 stage 1 tumor samples (of which 20 and 105, respectively, were female; technical replicates were removed, with retention of the most recently timestamped replicate). CNA data were obtained from Xenabrowser [20], opting for the gistic2 thresholded pipeline for gene-level CNA data (plotted in Fig. 5 and Supplementary Figs. S1–S4). HumanMethylation450 array probe data and an already processed KIRC htseq-based count file for DE analysis were obtained from Xenabrowser as well. As Xenabrowser recently replaced all htseq-based files by STAR-based files, we provide the former with this publication for completeness (TCGA-KIRC.htseq_counts.tsv). Table M1 provides references and download sources to all data described here.

**Table M1:** Table of used and created datasets, software, and websites

Datasets		
Data name	Reference	Source
GRCh38 TCGA KIRC RNA-seq BAM files	Grossman et al. [17]	https://portal.gdc.cancer.gov/analysis_page?app=Downloads
GRCh38.d1.vd1 Reference Sequence	Grossman et al. [17]	https://gdc.cancer.gov/about-data/gdc-data-processing/gdc-reference-files
Gene-level DNA copy number data (gistic2 thresholded)	Goldman et al. [20]	https://xenabrowser.net/datapages/?dataset=TCGA.KIRC.sampleMap%2FGistic2_CopyNumber_Gistic2_all_thresholded.by_genes&host=https%3A%2F%2Ftcga.xenahubs.net&removeHub=https%3A%2F%2Fxena.treehouse.gi.ucsc.edu%3A443
Illumina Human Methylation 450 data	Goldman et al. [20]	https://xenabrowser.net/datapages/?cohort=GDC%20TCGA%20Kidney%20Clear%20Cell%20Carcinoma%20(KIRC)&removeHub=https%3A%2F%2Fxena.treehouse.gi.ucsc.edu%3A443
Processed htseq gene count file for DE analysis	Goldman et al. [20]	TCGA-KIRC.htseq_counts.tsv: originally downloaded from Xenabrowser but no longer provided online; available from us upon request
Software		
Software name	Reference	Source
*maelstRom* source code	This article	https://github.com/Biobix/maelstRom
SAMtools	Li et al. [18]	https://www.htslib.org/
C++ boost library	Karlsson [70]	https://www.boost.org/
GNU scientific library	Gough [71]	https://www.gnu.org/software/gsl/
R *alabama* package	Varadhan [72]	https://CRAN.R-project.org/package=alabama
R *GenomicRanges* package	Lawrence et al. [76]	https://bioconductor.org/packages/release/bioc/html/GenomicRanges.html
R *EdgeR* package	Robinson et al. [3]	https://bioconductor.org/packages/release/bioc/html/edgeR.html
R *stats* package (part of R)	R Core Team [80]	https://www.R-project.org/
R *clusterProfiler* package	Wu et al. [78]	https://bioconductor.org/packages/release/bioc/html/clusterProfiler.html
Websites		
Site name	Reference	Source
*maelstRom* website	This article	https://biobix.github.io/maelstRom/
NCBI dbSNP database	Sherry et al. [19]	https://www.ncbi.nlm.nih.gov/snp/
MEXPRESS	Koch et al. [21]	https://mexpress.ugent.be/

#### BAM to SNP nucleotide counts

We used mpileup/bcftools from SAMtools [18] to infer SNP reference/variant counts from BAM files, after indexing if necessary, retaining only those with a minimal raw read depth of 10 in at least 1 sample and listed by dbSNP [19]. Nonuniquely mapped reads were filtered to reduce noise. Per-sample allelic counts (A/C/G/T) were written to count files together with dbSNP-ID and standard alleles (if available), as well as TCGA sample ID.

#### Determining a reference and variant allele

*maelstRom*’s beta-binomial models require input allelic counts to be restricted to only 2 alleles (here termed “reference” and “variant”). These are selected by assigning the most common allele per SNP (in terms of total count across all samples) as the reference and the second most common as the variant. If dbSNP provides standard alleles expected in human populations, this choice is restricted to those alleles only. In case of ties, the choice is made randomly. After retaining only reference and variant allele counts, only SNPs providing (nonzero) counts in at least 10 (out of 72) KIRC control samples were retained. This left 127,023 autosomal and 2,083 X chromosomal SNPs for further analyses.

#### DE and hypermethylation analysis

Gene-level count and promoter methylation data were obtained as described under the Data Acquisition section and subsequently processed into (FDR-corrected) DE and promoter *de novo* (hyper)methylation results, via *EdgeR*’s standard analysis pipeline following DE best practices [79] and the Fisher exact test (using *fisher.test* from the *stats* R package [80]) on the number of hypermethylated samples in controls and cases, respectively. A sample here was considered “hypermethylated” when featuring a methylation percentage (β-value) >20% for the, on average, most methylated CpG in each gene’s promotor region (using promoter annotation provided by MEXPRESS [21]) in its respective sample group (cases or controls). Note that the latter is conservative in a sense that the full promoter region is taken into account and that lower differences in the number of hypermethylated samples between cases and controls are expected. X chromosomal analyses used data originating from female samples only, for maximal comparability to dAD results. Results of these DE and differential methylation tests are depicted in Fig. 5 and Supplementary Figs. S1–S4. Genes without DE results (due to not appearing in the Xenabrowser-provided htseq-file) were removed from *maelstRom*’s dAD results, leading to complete results for 11,325 autosomal genes, and 291 X chromosome genes.

Similar to dAD *P* values, DE *P* values were FDR-corrected using the Benjamini–Hochberg procedure on the 11,325 autosomal and 291 X chromosomal genes separately. Differential promoter hypermethylation *P* values, which were not used for results filtering or follow-up analyses but are merely exploratively plotted in Fig. 5 and Supplementary Figs. S1–S4, were not corrected for multiple testing.

## Availability of Supporting Source Code and Requirements

Project name: *maelstRom*

Project homepage: https://github.com/Biobix/maelstRom

Operating system(s): e.g., Platform independent

Programming language: R (v4.0.2 or higher), C, C++

Other requirements: gmp v6.1.2 or higher, mpfr v4.0.1 or higher

License: APGL-3.0 license

## Supplementary Material

giaf125_Supplemental_Files

giaf125_Authors_Response_To_Reviewer_Comments_Original_Submission

giaf125_GIGA-D-25-00102_Original_Submission

giaf125_GIGA-D-25-00102_Revision_1

giaf125_Reviewer_1_Report_Original_Submission Anelia Horvath -- 4/15/2025

giaf125_Reviewer_2_Report_Revision_1 Vasyl Zhabotynsky -- 9/5/2025

giaf125_Revision_2_Report_Original_Submission Vasyl Zhabotynsky -- 5/5/2025

## Data Availability

The sequencing data (bam files) underlying this article are available from the GDC data portal [17] under the project identifier “TCGA-KIRC.” These files were aligned on the “GRCh38.d1.vd1” Reference Sequence, available from the same GDC data portal. Additional gene-level DNA copy number data and Illumina Human Methylation 450 data underlying this article are available on the Xenabrowser portal [20], under the respective dataset IDs “TCGA.KIRC.sampleMap/Gistic2_CopyNumber_Gistic2_all_thresholded.by_genes” and “TCGA-KIRC.methylation450.tsv.” The processed htseq gene count file for DE analyses underlying this article (with ID “TCGA-KIRC.htseq_counts.tsv”) was also downloaded for Xenabrowser but is no longer available online; it is available through the *GigaScience* repository, GigaDB [81], together with other supporting scripts and data.

## Additional Files

**Supplementary Table S1**. *maelstRom* dAD and DE results for the PCDH gene cluster, ordered according to chromosomal location, along with some surrounding genes. Included are per-sample sequencing coverages in both controls and cases (**cov_control_, cov_case_**; both calculated as the mean [across SNPs] of median sample coverages), results of the dAD fit (**ρ_control_, ρ_case_, p_dAD_**), and a DE analysis (**log_2_FC, p_DE_**). dAD, as well as ρ_case_, is generally high across chromosome 5q in KIRC due to very common CNA gain, as is apparent from the genes surrounding the PCDH gene cluster. Nevertheless, these do not reach the enormous ρ_case_, as observed across PCDHs in the cluster (not counting the C-type PCDHGC3, which showcases unremarkable ρ_case_ similar to surrounding genes, likely indicative of its non-RME, biallelic expression). Such extreme ρ_case_ is not even observed for TAF7, which is located right in between the β- and γ-PCDHs. Also noteworthy is the generally low coverage observed across PCDHs (again, with the exception of likely constitutively expressed PCDHGC3), which is possibly indicative of their stochastic expression (see main text Results). At the end of the table lies a “regular” PCDH not located in the cluster, which does, however, not behave similar to the cluster itself regarding dAD or expression in general.

**Supplementary Table S2**. Overrepresented biological process (BP) Gene Ontology terms among DE genes. Resulting GO terms were filtered on redundancy, excluding terms if their involved genes showed at least 60% overlap with a more significant GO term; Methods lists significance and other filter criteria.

**Supplementary Table S3**. Overrepresented cellular component (CC) Gene Ontology terms among dAD, dAD-and-DE, and DE genes. Resulting GO terms were filtered on redundancy, excluding terms if their involved genes showed at least 60% overlap with a more significant GO term; Methods lists significance and other filter criteria.

**Supplementary Table S4**. Overrepresented molecular function (MF) Gene Ontology terms among dAD, dAD-and-DE, and DE genes. Resulting GO terms were filtered on redundancy, excluding terms if their involved genes showed at least 60% overlap with a more significant GO term; Methods lists significance and other filter criteria.

**Supplementary Fig. S1**. Chromosome-wide gene-level (d)AD results, as $\rho $, for chromosomes 1 through 6. Top plots within each panel display ${\rho }_{\textit{control}}$ in black and ${\rho }_{\textit{case}}$ color-coded according to statistical significance of differential AD (FDR-corrected ${p}_{dAD}$; turns from blue to red at 0.05), together with DE results as $lo{g}_2( {\textit{Fold}\ \textit{Change}} )$. Bottom panels display *P* values testing for tumor promoter hypermethylation ($- log( {{p}_{\textit{meth}}} );$ raw *P* value with orange dotted line at ${p}_{\textit{meth}}$ = 0.05) and the average copy number loss and gain in tumor samples. All measures are visualized as rolling medians (window size of 15 genes) to emphasize genomic regions rather than individual genes.

**Supplementary Fig. S2**. Chromosome-wide gene-level (d)AD results, as $\rho $, for chromosomes 7 through 12. Top plots within each panel display ${\rho }_{\textit{control}}$ in black and ${\rho }_{\textit{case}}$ color-coded according to statistical significance of differential AD (FDR-corrected ${p}_{dAD}$; turns from blue to red at 0.05), together with DE results as $lo{g}_2( {\textit{Fold}\ \textit{Change}} )$. Bottom panels display *P* values testing for tumor promoter hypermethylation ($- log( {{p}_{\textit{meth}}} );$ raw *P* value with orange dotted line at ${p}_{\textit{meth}}$ = 0.05) and the average copy number loss and gain in tumor samples. All measures are visualized as rolling medians (window size of 15 genes) to emphasize genomic regions rather than individual genes.

**Supplementary Fig. S3**. Chromosome-wide gene-level (d)AD results, as $\rho $, for chromosomes 13 through 18. Note that chromosomes 13, 14, and 15 are 3 (out of 5) acrocentric chromosomes, which have short, extremely gene-poor, rDNA-containing p-arms. Thus, no gene-level data exist for these regions. Top plots within each panel display ${\rho }_{\textit{control}}$ in black and ${\rho }_{\textit{case}}$ color-coded according to statistical significance of differential AD (FDR-corrected ${p}_{dAD}$; turns from blue to red at 0.05), together with DE results as $lo{g}_2( {\textit{Fold}\ \textit{Change}} )$. Bottom panels display *P* values testing for tumor promoter hypermethylation ($- log( {{p}_{\textit{meth}}} );$ raw *P* value with orange dotted line at ${p}_{\textit{meth}}$ = 0.05) and the average copy number loss and gain in tumor samples. All measures are visualized as rolling medians (window size of 15 genes) to emphasize genomic regions rather than individual genes.

**Supplementary Fig. S4**. Chromosome-wide gene-level (d)AD results, as $\rho $, for chromosomes 19 through 22. Note that chromosomes 21 and 22 are 2 (out of 5) acrocentric chromosomes, which have short, extremely gene-poor, rDNA-containing p-arms. Thus, no gene-level data exist for these regions. Top plots within each panel display ${\rho }_{\textit{control}}$ in black and ${\rho }_{\textit{case}}$ color-coded according to statistical significance of differential AD (FDR-corrected ${p}_{dAD}$; turns from blue to red at 0.05), together with DE results as $lo{g}_2( {\textit{Fold}\ \textit{Change}} )$. Bottom panels display *P* values testing for tumor promoter hypermethylation ($- log( {{p}_{\textit{meth}}} );$ raw *P* value with orange dotted line at ${p}_{\textit{meth}}$ = 0.05) and the average copy number loss and gain in tumor samples. All measures are visualized as rolling medians (window size of 15 genes) to emphasize genomic regions rather than individual genes.

## Abbreviations

AB: allelic bias; AD: allelic dispersion; ASE: allele-specific expression; BFGS: Broyden–Fletcher–Goldfarb–Shanno algorithm; BP: biological process; CC: cellular component; CDF: cumulative distribution function; *cis*-eQTL: *cis*-expression quantitative trait locus; CMF: cumulative mass function; CNA: copy number alteration; dAD: differential allelic dispersion; DE: differential expression; EM: expectation–maximization; FDR: false discovery rate; GO: Gene Ontology; HWE: Hardy–Weinberg equilibrium; KIRC: kidney renal clear cell carcinoma; LRT: likelihood ratio test; MF: molecular function; ORA: overrepresentation analysis; PCDH: protocadherin; PMF: probability mass function; RME: random monoallelic expression; RNA-seq: RNA sequencing; SE: sequencing error; SNP: single-nucleotide polymorphism; TCGA: The Cancer Genome Atlas; XCI: X chromosome inactivation.
