# Supplementary material for: Population-level allelic dispersion modeling by maelstRom yields genome-wide maps of allele-specific dysregulation during early carcinogenesis
Source: Gigascience. 2025 Nov 4;14:giaf125. doi: 10.1093/gigascience/giaf125 (PMC12585351; doi:10.1093/gigascience/giaf125)
Supplement: giaf125_Supplemental_Files [file giaf125_supplemental_files.zip › maelstRom_SupplementaryFiguresAndTables.docx]

| **Gene** | **cov_control_** | **cov_case_** | **ρ_control_** | **ρ_case_** | **p_dAD_** | **log_2_FC** | **p_DE_** |
| --- | --- | --- | --- | --- | --- | --- | --- |
| SLC35A4 | 77 | 75 | <0.001 | 0.033 | 1.89E-03 | 0.017 | 7.42E-01 |
| TMCO6 | 38 | 51 | 0.005 | 0.054 | 3.55E-06 | 0.792 | 2.25E-27 |
| IK | 200 | 224 | 0.002 | 0.033 | 7.42E-05 | 0.321 | 3.21E-11 |
| WDR55 | 59 | 72 | 0.003 | 0.055 | 4.69E-08 | 0.483 | 1.35E-18 |
| ZMAT2 | 142 | 140 | <0.001 | 0.050 | 4.53E-12 | 0.100 | 8.89E-02 |
| PCDHA4 | 5 | 4 | 0.107 | 0.626 | 1.66E-05 | 0.107 | 6.86E-01 |
| PCDHB2 | 4 | 7 | 0.000 | 0.597 | 4.81E-02 | 1.015 | 2.60E-06 |
| PCDHB3 | 9 | 6 | 0.095 | 0.475 | 1.16E-12 | 0.227 | 2.40E-01 |
| PCDHB4 | 10 | 10 | 0.042 | 0.212 | 6.30E-03 | 0.333 | 3.45E-02 |
| PCDHB5 | 16 | 7 | 0.055 | 0.538 | 3.50E-04 | -0.514 | 1.27E-02 |
| PCDHB6 | 4 | 9 | 0.005 | 0.503 | 7.51E-10 | 1.804 | 5.97E-15 |
| PCDHB7 | 7 | 9 | 0.090 | 0.300 | 1.16E-06 | 0.422 | 7.90E-03 |
| PCDHB16 | 15 | 19 | 0.064 | 0.307 | 5.51E-09 | 0.941 | 9.66E-08 |
| PCDHB9 | 5 | 12 | 0.015 | 0.267 | 5.48E-05 | 1.399 | 8.31E-20 |
| PCDHB10 | 5 | 11 | 0.035 | 0.185 | 1.32E-01 | 1.651 | 9.50E-30 |
| PCDHB11 | 8 | 8 | 0.053 | 0.401 | 6.36E-08 | 0.592 | 4.39E-03 |
| PCDHB12 | 7 | 11 | <0.001 | 0.183 | 6.36E-01 | 0.895 | 3.77E-12 |
| PCDHB13 | 6 | 8 | 0.114 | 0.198 | 7.37E-01 | 0.519 | 1.58E-05 |
| PCDHB14 | 8 | 12 | 0.085 | 0.196 | 3.57E-03 | 1.295 | 6.55E-20 |
| PCDHB15 | 7 | 11 | 0.096 | 0.262 | 2.93E-02 | 0.966 | 6.39E-08 |
| TAF7 | 275 | 290 | 0.001 | 0.055 | 2.31E-08 | 0.063 | 3.01E-01 |
| PCDHGA2 | 10 | 4 | 0.018 | 0.221 | 6.18E-02 | -0.560 | 3.08E-04 |
| PCDHGA4 | 5 | 5 | <0.001 | 0.405 | 1.22E-01 | 0.189 | 2.63E-01 |
| PCDHGB2 | 4 | 6 | 0.076 | 0.466 | 8.51E-02 | 1.739 | 7.97E-12 |
| PCDHGB5 | 14 | 5 | 0.002 | 0.421 | 1.06E-07 | -0.601 | 2.02E-03 |
| PCDHGA9 | 7 | 7 | 0.067 | 0.414 | 3.12E-06 | 0.969 | 2.78E-05 |
| PCDHGB6 | 9 | 7 | 0.025 | 0.242 | 6.60E-04 | 0.506 | 2.30E-03 |
| PCDHGB7 | 10 | 14 | 0.116 | 0.396 | 7.88E-05 | 1.320 | 6.33E-10 |
| PCDHGC3 | 33 | 73 | 0.036 | 0.049 | 6.81E-01 | 1.146 | 7.00E-19 |
| HDAC3 | 95 | 112 | 0.017 | 0.051 | 1.45E-02 | 0.460 | 2.79E-21 |
| RELL2 | 11 | 14 | 0.015 | 0.026 | 4.25E-01 | 0.156 | 2.52E-01 |
| FCHSD1 | 8 | 15 | 0.044 | 0.055 | 8.52E-01 | 1.137 | 2.07E-24 |
| ARAP3 | 22 | 38 | 0.008 | 0.006 | 7.75E-01 | 1.072 | 3.89E-19 |
| PCDH1 | 98 | 48 | 0.004 | 0.021 | 1.54E-01 | -0.851 | 1.22E-15 |

**Supplementary Tables**

**Table S1: maelstRom dAD and DE results for the PCDH gene cluster, ordered according to chromosomal location, along with some surrounding genes.** Included are per-sample sequencing coverages in both controls and cases (**cov_control_, cov_case_**; both calculated as the mean (across SNPs) of median sample coverages), results of the dAD fit (**ρ_control,_ ρ_case,_ p_dAD_**) and a DE analysis (**log_2_FC, p_DE_**). dAD, and ρ_case_, is generally high across chromosome 5q in KIRC due to very common CNA gain, as is apparent from the genes surrounding the PCDH gene cluster. Nevertheless, these do not reach the enormous ρ_case_ as observed across PCDHs in the cluster (not counting the C-type PCDHGC3, which showcases unremarkable ρ_case_ similar to surrounding genes, likely inidicative of its non-RME, biallelic expression). Such extreme ρ_case_ is not even observed for TAF7, which is located right in between the β- and γ-PCDHs. Also noteworthy is the generally low coverage observed across PCDHs (again, with the exception of likely constitutively expressed PCDHGC3), which is possibly indicative of their stochastic expression (see main text Results). At the end of the table lies a “regular” PCDH not located in the cluster; which does, however, not behave similar to the cluster itself regarding dAD, or expression in general.

Included are results of the dAD fit (**ρ_control,_ ρ_case,_ p_dAD_**), DE analysis (**log_2_FC, p_DE_**) and sample-level correlations as described in Section 2.3 with corresponding p-values (**Corr_AD-EX -_ p_AD-Meth_**). The final column (**Ref**) provides high-impact references indicating each gene in cancers, with the exception of ALPK3 (*), for which a publication on its paralog ALPK2 is provided instead (Section 3.3).

**Table S2: Overrepresented biological process (BP) gene ontology terms amongst DE genes.** Resulting GO terms were filtered on redundancy, excluding terms if their involved genes showed at least 60% overlap with a more significant GO term; Methods lists significance- and other filter criteria.

| **BP GO-terms enriched for significant DE (2207/11325 genes)** | | | |
| --- | --- | --- | --- |
| **Description** | **p_FDR_** | **Description** | **p_FDR_** |
| adaptive immune response | 2.81E-29 | cellular defense response | 1.33E-04 |
| leukocyte migration | 3.75E-25 | negative regulation of transport | 1.44E-04 |
| regulation of cell adhesion | 1.68E-24 | trans-synaptic signaling | 1.90E-04 |
| regulation of cell activation | 5.27E-23 | multicellular organismal homeostasis | 2.11E-04 |
| humoral immune response | 9.13E-22 | response to nutrient | 4.33E-04 |
| extracellular matrix organization | 5.37E-21 | response to hypoxia | 4.62E-04 |
| inflammatory response | 3.20E-20 | small GTPase mediated signal transduction | 5.11E-04 |
| chemotaxis | 3.37E-20 | regulation of GTPase activity | 5.41E-04 |
| response to bacterium | 4.00E-18 | small molecule catabolic process | 5.57E-04 |
| positive regulation of locomotion | 2.11E-16 | regulation of protein kinase B signaling | 5.78E-04 |
| angiogenesis | 2.60E-16 | cytolysis | 6.03E-04 |
| G protein-coupled receptor signaling pathway | 9.93E-15 | response to mechanical stimulus | 7.61E-04 |
| negative regulation of response to external stimulus | 3.18E-12 | gland development | 7.85E-04 |
| cytokine-mediated signaling pathway | 1.11E-11 | negative regulation of cell differentiation | 8.15E-04 |
| immune response-regulating signaling pathway | 1.46E-11 | collagen metabolic process | 8.61E-04 |
| phagocytosis | 1.55E-11 | mesenchymal cell differentiation | 8.99E-04 |
| cell activation involved in immune response | 3.33E-11 | connective tissue development | 1.10E-03 |
| leukocyte differentiation | 3.39E-11 | response to drug | 1.10E-03 |
| divalent inorganic cation homeostasis | 8.69E-11 | organic hydroxy compound metabolic process | 1.12E-03 |
| cytokine production | 9.36E-11 | organic hydroxy compound transport | 1.14E-03 |
| wound healing | 1.05E-10 | activation of phospholipase C activity | 1.37E-03 |
| nervous system process | 3.87E-10 | anatomical structure maturation | 1.49E-03 |
| positive regulation of response to external stimulus | 1.60E-09 | regulation of hormone levels | 1.50E-03 |
| urogenital system development | 1.78E-09 | response to activity | 1.58E-03 |
| regulation of ion transport | 1.86E-09 | regulation of vesicle-mediated transport | 1.67E-03 |
| anion transport | 1.90E-09 | regulation of membrane potential | 1.79E-03 |
| cell-cell adhesion via plasma-membrane adhesion molecules | 2.84E-09 | endothelium development | 1.97E-03 |
| ERK1 and ERK2 cascade | 1.24E-08 | segmentation | 1.99E-03 |
| circulatory system process | 2.41E-08 | striated muscle tissue development | 2.31E-03 |
| cell junction organization | 3.34E-08 | aging | 2.31E-03 |
| cell recognition | 4.19E-08 | positive regulation of striated muscle cell differentiation | 2.33E-03 |
| peptidyl-tyrosine phosphorylation | 1.05E-07 | regulation of epithelial cell apoptotic process | 2.42E-03 |
| cell killing | 1.57E-07 | regulation of signaling receptor activity | 2.86E-03 |
| second-messenger-mediated signaling | 1.66E-07 | detection of stimulus | 2.96E-03 |
| regulation of secretion | 1.77E-07 | negative regulation of endopeptidase activity | 3.00E-03 |
| negative regulation of cellular component movement | 2.53E-07 | multicellular organismal response to stress | 3.06E-03 |
| branching morphogenesis of an epithelial tube | 2.60E-07 | positive regulation of lipid localization | 3.21E-03 |
| monocarboxylic acid metabolic process | 5.21E-07 | response to ethanol | 3.28E-03 |
| inorganic ion transmembrane transport | 7.30E-07 | skin development | 3.31E-03 |
| endocytosis | 9.21E-07 | renal system process | 3.59E-03 |
| import into cell | 9.64E-07 | regulation of smooth muscle cell proliferation | 3.68E-03 |
| transmembrane receptor protein tyrosine kinase signaling pathway | 1.41E-06 | multi-multicellular organism process | 3.69E-03 |
| epithelial cell proliferation | 1.50E-06 | cell morphogenesis involved in differentiation | 3.83E-03 |
| tissue remodeling | 1.65E-06 | zymogen activation | 4.15E-03 |
| negative regulation of cell population proliferation | 1.92E-06 | superoxide metabolic process | 4.52E-03 |
| regulation of protein kinase C signaling | 2.74E-06 |  |  |

**Table S3: Overrepresented cellular component (CC) gene ontology terms amongst dAD, dAD-and-DE, and DE genes.** Resulting GO terms were filtered on redundancy, excluding terms if their involved genes showed at least 60% overlap with a more significant GO term; Methods lists significance- and other filter criteria.

| **CC GO-terms enriched for significant dAD (2142/11325 genes)** | |
| --- | --- |
| **Description** | **p_FDR_** |
| mitochondrial matrix | 9.92E-12 |
| mitochondrial inner membrane | 7.60E-09 |
| primary lysosome | 1.75E-06 |
| oxidoreductase complex | 5.98E-05 |
| vacuolar lumen | 1.40E-04 |
| **CC GO-terms enriched for significant dAD and DE (942/11325 genes)** | |
| **Description** | **p_FDR_** |
| mitochondrial matrix | 2.73E-13 |
| apical plasma membrane | 6.16E-07 |
| basolateral plasma membrane | 8.45E-07 |
| **CC GO-terms enriched for significant DE (2207/11325 genes)** | |
| **Description** | **p_FDR_** |
| cell surface | 3.11E-38 |
| extracellular matrix | 3.70E-31 |
| plasma membrane protein complex | 1.28E-15 |
| immunoglobulin complex | 1.39E-13 |
| apical part of cell | 6.63E-13 |
| receptor complex | 1.04E-10 |
| basolateral plasma membrane | 1.23E-10 |
| secretory granule membrane | 5.42E-10 |
| membrane microdomain | 2.09E-07 |
| endoplasmic reticulum lumen | 3.20E-07 |
| cell-cell junction | 2.18E-06 |
| immunological synapse | 1.29E-05 |
| endocytic vesicle membrane | 2.97E-05 |
| microvillus | 4.09E-05 |
| blood microparticle | 5.69E-05 |
| cell projection membrane | 8.92E-05 |
| sarcolemma | 1.45E-04 |
| postsynaptic membrane | 3.84E-04 |
| platelet alpha granule | 6.36E-04 |

**Table S4: Overrepresented molecular function (MF) gene ontology terms amongst dAD, dAD-and-DE, and DE genes.** Resulting GO terms were filtered on redundancy, excluding terms if their involved genes showed at least 60% overlap with a more significant GO term; Methods lists significance- and other filter criteria.

| **MF GO-terms enriched for significant dAD (2142/11325 genes)** | |
| --- | --- |
| **Description** | **p_FDR_** |
| oxidoreductase activity | 5.20E-10 |
| lyase activity | 4.32E-05 |
| vitamin binding | 5.08E-05 |
| **MF GO-terms enriched for significant dAD and DE (942/11325 genes)** | |
| **Description** | **p_FDR_** |
| oxidoreductase activity | 4.19E-12 |
| hydro-lyase activity | 3.92E-08 |
| vitamin binding | 2.71E-06 |
| inorganic molecular entity transmembrane transporter activity | 7.20E-06 |
| fatty acid binding | 1.68E-04 |
| **MF GO-terms enriched for significant DE (2207/11325 genes)** | |
| **Description** | **p_FDR_** |
| signaling receptor activity | 6.63E-37 |
| antigen binding | 1.12E-22 |
| extracellular matrix structural constituent | 5.44E-17 |
| calcium ion binding | 4.80E-15 |
| receptor regulator activity | 1.64E-13 |
| inorganic molecular entity transmembrane transporter activity | 6.94E-11 |
| glycosaminoglycan binding | 9.49E-10 |
| carbohydrate binding | 1.17E-09 |
| carboxylic acid binding | 2.09E-06 |
| serine hydrolase activity | 2.43E-06 |
| MHC protein binding | 2.61E-06 |
| iron ion binding | 3.00E-06 |
| amyloid-beta binding | 3.66E-06 |
| growth factor binding | 4.22E-06 |
| G protein-coupled receptor binding | 5.14E-06 |
| integrin binding | 1.20E-05 |
| cargo receptor activity | 1.54E-05 |
| metalloendopeptidase activity | 6.45E-05 |
| GTPase regulator activity | 1.28E-04 |
| heme binding | 1.49E-04 |
| oxidoreductase activity, acting on CH-OH group of donors | 2.49E-04 |
| extracellular matrix binding | 3.15E-04 |
| transferase activity, transferring nitrogenous groups | 3.42E-04 |
| xenobiotic transmembrane transporter activity | 3.94E-04 |
| collagen binding | 4.43E-04 |
| peptidase regulator activity | 5.89E-04 |
| lipid transporter activity | 8.83E-04 |

**Supplementary Figures**


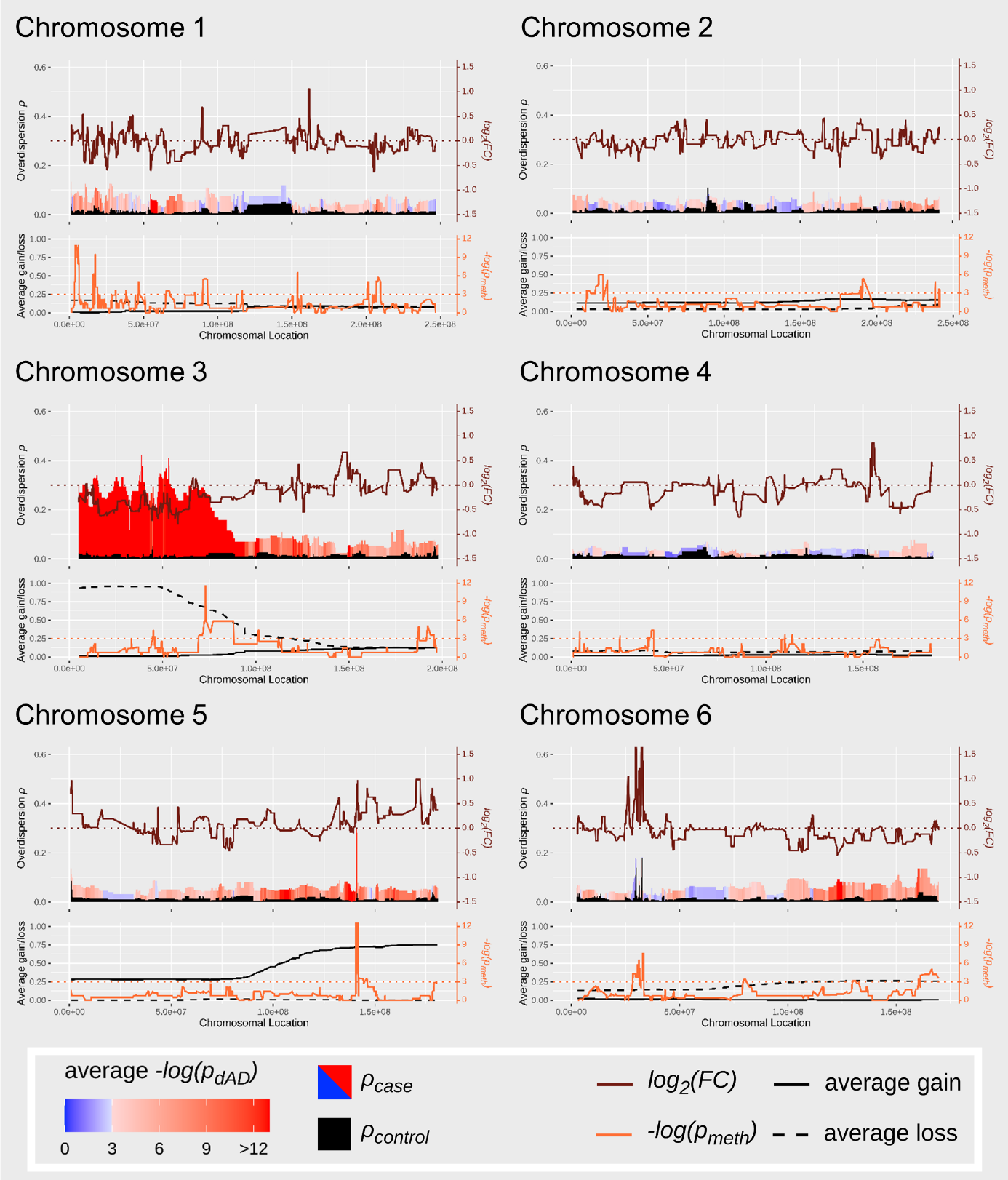


**Figure S1: Chromosome-wide gene-level (d)AD results, as** $\boldsymbol{\rho}$**, for chromosomes 1 through 6.** Top plots within each panel display $\rho_{control}$ in black and $\rho_{case}$ color-coded according to statistical significance of differential AD (FDR-corrected $p_{dAD}$; turns from blue to red at 0.05), together with DE results as ${log}_{2}\left( Fold Change \right)$. Bottom panels display p-values testing for tumor promoter hypermethylation ($-log\left( p_{meth} \right);$ raw p-value with orange dotted line at $p_{meth}$=0.05) and the average copy number loss and gain in tumor samples. All measures are visualized as rolling medians (window size of 15 genes) to emphasize genomic regions rather than individual genes.

**
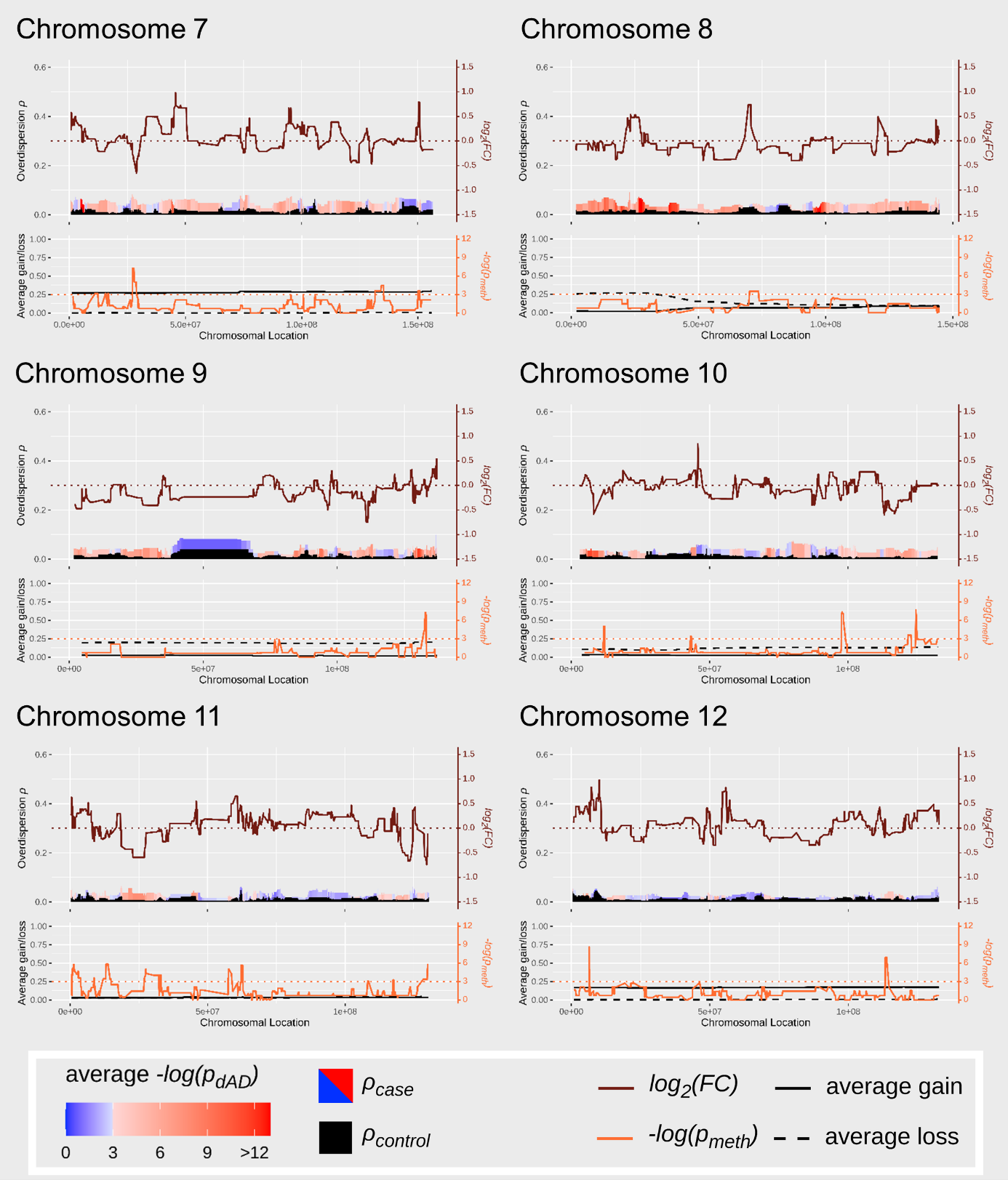
**

**Figure S2: Chromosome-wide gene-level (d)AD results, as** $\boldsymbol{\rho}$**, for chromosomes 7 through 12.** Top plots within each panel display $\rho_{control}$ in black and $\rho_{case}$ color-coded according to statistical significance of differential AD (FDR-corrected $p_{dAD}$; turns from blue to red at 0.05), together with DE results as ${log}_{2}\left( Fold Change \right)$. Bottom panels display p-values testing for tumor promoter hypermethylation ($-log\left( p_{meth} \right);$ raw p-value with orange dotted line at $p_{meth}$=0.05) and the average copy number loss and gain in tumor samples. All measures are visualized as rolling medians (window size of 15 genes) to emphasize genomic regions rather than individual genes.

**
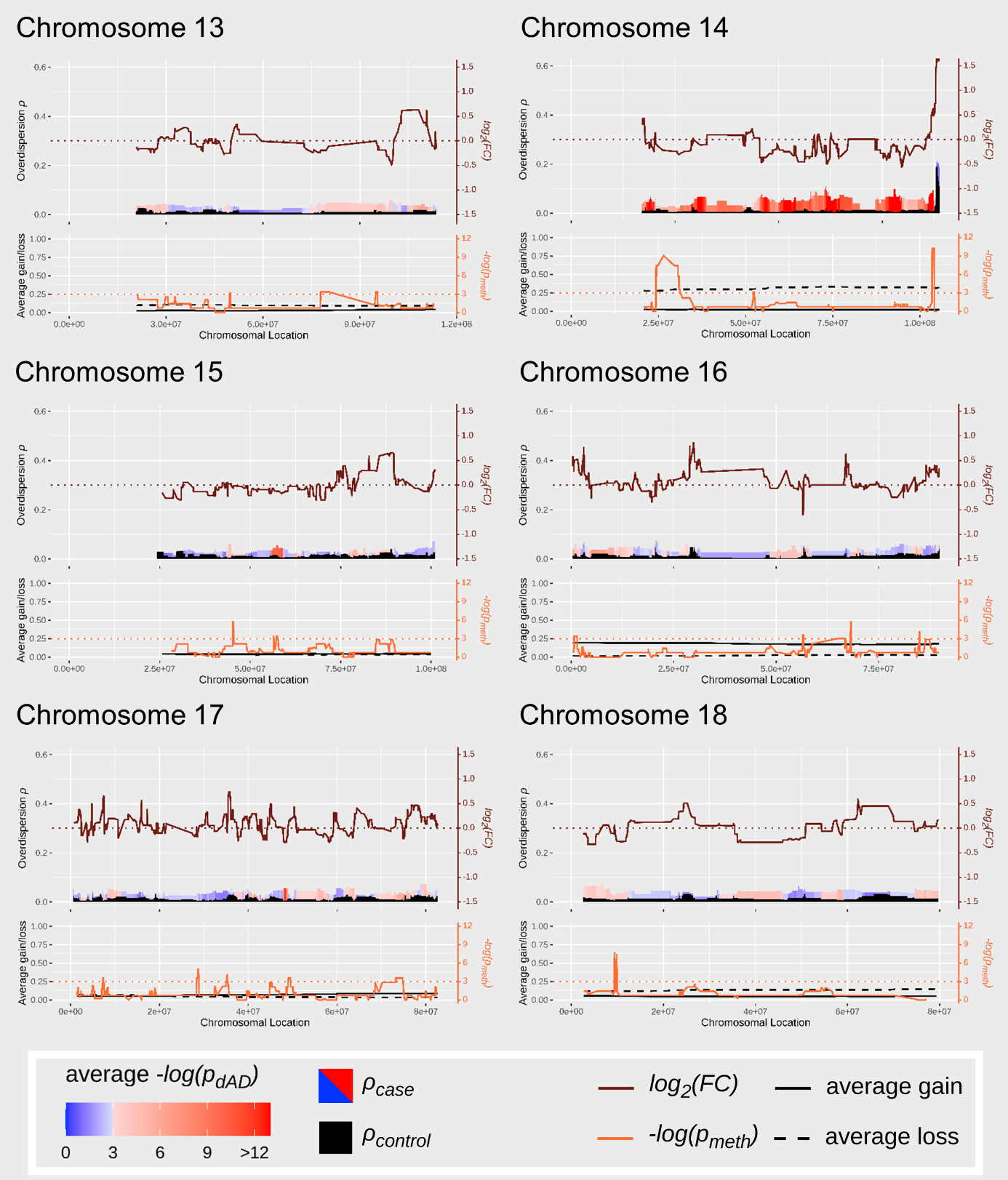
**

**Figure S3: Chromosome-wide gene-level (d)AD results, as** $\boldsymbol{\rho}$**, for chromosomes 13 through 18.** Note that chromosomes 13, 14, and 15 are three (out of five) acrocentric chromosomes, which have short, extremely gene-poor, rDNA-containing p-arms. Thus, no gene-level data exists for these regions. Top plots within each panel display $\rho_{control}$ in black and $\rho_{case}$ color-coded according to statistical significance of differential AD (FDR-corrected $p_{dAD}$; turns from blue to red at 0.05), together with DE results as ${log}_{2}\left( Fold Change \right)$. Bottom panels display p-values testing for tumor promoter hypermethylation ($-log\left( p_{meth} \right);$ raw p-value with orange dotted line at $p_{meth}$=0.05) and the average copy number loss and gain in tumor samples. All measures are visualized as rolling medians (window size of 15 genes) to emphasize genomic regions rather than individual genes.


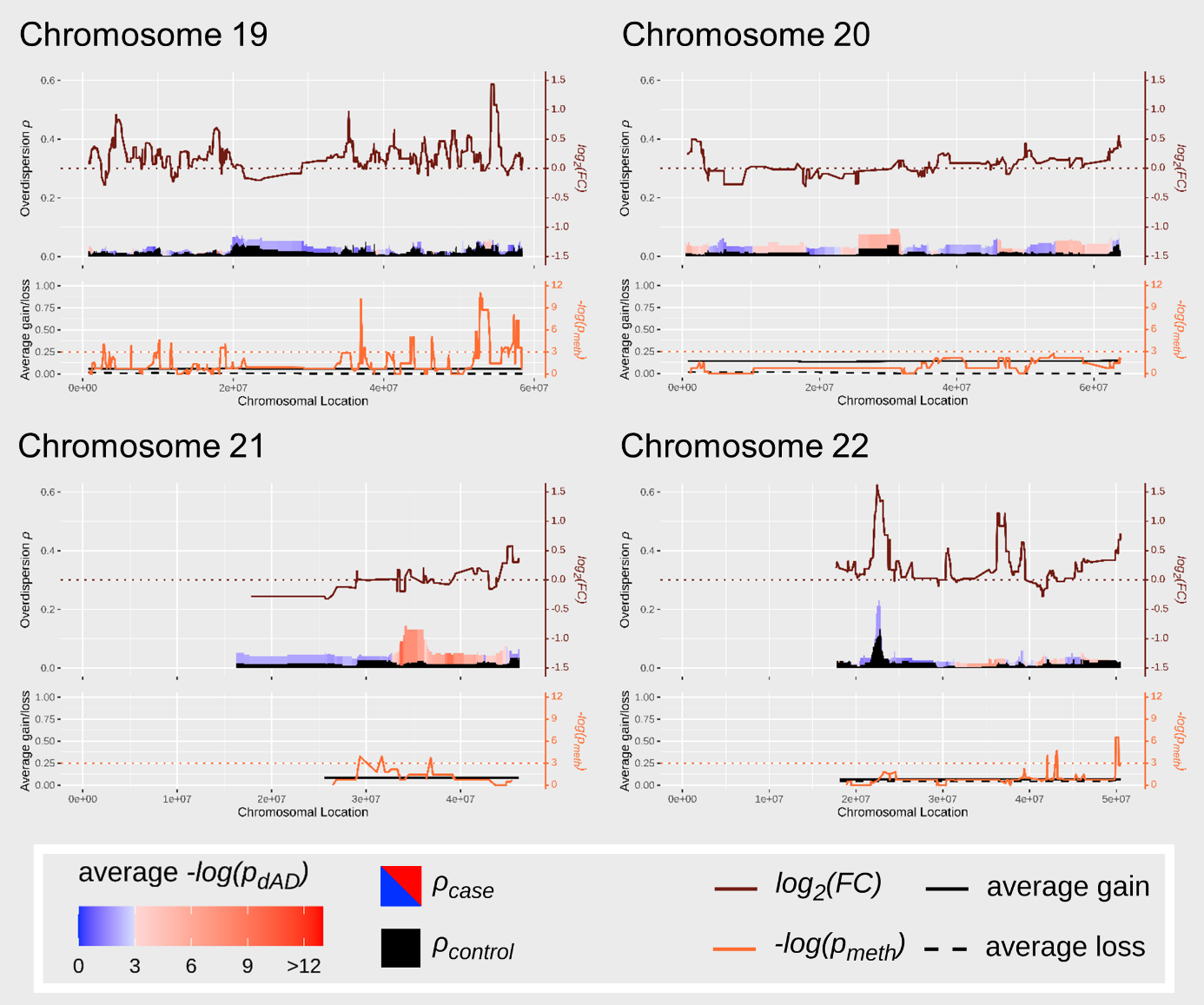


**Figure S4: Chromosome-wide gene-level (d)AD results, as** $\boldsymbol{\rho}$**, for chromosomes 19 through 22.** Note that chromosomes 21 and 22 are two (out of five) acrocentric chromosomes, which have short, extremely gene-poor, rDNA-containing p-arms. Thus, no gene-level data exists for these regions. Top plots within each panel display $\rho_{control}$ in black and $\rho_{case}$ color-coded according to statistical significance of differential AD (FDR-corrected $p_{dAD}$; turns from blue to red at 0.05), together with DE results as ${log}_{2}\left( Fold Change \right)$. Bottom panels display p-values testing for tumor promoter hypermethylation ($-log\left( p_{meth} \right);$ raw p-value with orange dotted line at $p_{meth}$=0.05) and the average copy number loss and gain in tumor samples. All measures are visualized as rolling medians (window size of 15 genes) to emphasize genomic regions rather than individual genes.
