# Supplementary material for: Population-level allelic dispersion modeling by maelstRom yields genome-wide maps of allele-specific dysregulation during early carcinogenesis
Source: Gigascience. 2025 Nov 4;14:giaf125. doi: 10.1093/gigascience/giaf125 (PMC12585351; doi:10.1093/gigascience/giaf125)
Supplement: giaf125_Authors_Response_To_Reviewer_Comments_Original_Submission [file giaf125_authors_response_to_reviewer_comments_original_submission.pdf]

Author's Response To Reviewer Comments

First off, we would like to thank both reviewers for their insightful feedback. Having incorporated their suggestions in several parts of our main text has certainly improved its clarity, and even revealed some new results. As we could not, however, completely accommodate all of their suggestions, we have provided a response to their remarks below (containing whether and how these were addressed, and our thoughts on the matter or some extra information not included in the main text where applicable). Please do not hesitate to contact us with any follow-up responses or requests, or remaining reservations, you may have following this revision.

> R1: It would be highly informative to see a genome-wide overview of dAD using circos plots or comparable genome-scale visualizations, either in the main figures or as supplementary material.

We have incorporated this remark into the new Figure 6 of our revised manuscript. This supplants Supplementary Figures S1-S4 at several parts of the main text. In addition, due to the circos plot's specific layout, we actually noticed an RME region that went unnoticed in our previous figures, the IGKV gene cluster, and have incorporated this new result into the main text (along with other new and notable observations in this circos plot). Given these improvements to both results visualization and interpretation, we thank the reviewer for this great suggestion.

> R1: The authors should clarify the intended application of their population-based dAD method. Is maelstRom meant to be run once per cohort or cancer type to discover candidate early dysregulated loci, or is the goal to eventually support individual-level predictions (e.g., for biomarker development or risk stratification)? If the latter, it is critical to explain how population-derived dispersion parameters could be applied to classify or interpret single samples. At present, the manuscript does not clearly distinguish between discovery-based cohort-level analysis and translational utility at the patient level, and this distinction is essential for assessing the method's broader relevance. […] What exactly is the application of this population-based method? Is it meant to be run once per cohort/cancer type and then used in individual sample risk prediction? The authors need to clarify better this.

A discussion of maelstRom's general biological utility was indeed rather limited in the previous version of our manuscript, and an (in hindsight) careless mention of biomarkers in our introduction no doubt contributed to confusion about maelstRom's relevant use-cases (population-level studies or individual-specific biomarkers? It is, indeed, the former and not – directly – the latter). We have thus removed this carelessness from our introduction, and included a new biological subsection in our discussion to address this remark.

> R1: The authors provide a detailed yet ultimately unconvincing rationale for limiting their study to renal clear cell carcinoma (RCC). While RCC is a compelling model due to its early and clonally stable allele-specific copy number alterations (e.g., 3p loss), it is not unique in this respect. Several other cancers—including breast (BRCA), lung adenocarcinoma (LUAD), prostate (PRAD), colon (COAD), and glioblastoma (GBM)—exhibit similarly well-characterized early events such as allelic copy number changes, promoter methylation, and epigenetic silencing, many of which are clonal and mitotically maintained. Applying maelstRom to these additional tumor types would not only provide critical validation of the method's generalizability but would also illuminate tissue-specific versus pan-cancer patterns of early allele-specific dysregulation.

It is, of course, true that a pan-cancer application of maelstRom would both provide additional – potentially very interesting – results, and deepen understanding of our current results. However, in our study, we aimed to first and foremost establish the underlying (biological) rationale, the methodologies, and results

exploration and interpretation of the maelstRom software package, the latter part of which KIRC was a compelling and focused case study for (for reasons mentioned by the reviewer). This is also the reason we submitted our manuscript as a technical note, being primarily intended as a methodological introduction to the technique supported by a comprehensible yet relevant illustration (which could, indeed, have been another cancer), rather than aiming for an in-depth pan-cancer study. We thus appreciate the reviewer’s interest in maelstRom’’s results in such a comprehensive follow-up study, but believe including this in the current – already very information-dense and technical – paper would be too much, and even distract from the separate cancer-biology focus such a study would entail and deserve in publication. That being said, we certainly aim to tackle this suggestion in follow-up research, though a study of said scale entails considerable effort (we estimate a year or longer) to provide the depth and quality we strive for. This is in itself a more practical reason why we cannot fully address the remark in this revision’s timeframe. We do, however, acknowledge its expected merits, as underlined by the reviewer, in the (previously mentioned) new biological discussion-subsection of our revised manuscript.

> R1: Benchmarking: maelstRom is positioned as unique, yet it would be valuable to compare its performance (e.g., runtime, sensitivity, specificity) to existing ASE frameworks (e.g., MBASED, QuASAR, RASQUAL) where applicable.

We understand the desire to have some benchmarking to existing ASE-tools, and extensively searched for relevant points of comparison among existing software tools ourselves. However, maelstRom simply provides a hitherto unexplored analysis type which is very distinct from the (cis-)eQTL analyses the here mentioned (and other) methodologies provide. A direct comparison of runtime would thus be irrelevant. The reviewer acknowledges this with a final “where applicable”, and we hope this response clears up why something of this kind was not included in our study.

>R1: The authors infer that dAD identifies putative causal, upstream events based on its ability to capture clonally maintained allele-specific dysregulation across a population, its co-localization with known early driver events (e.g., 3p loss, XCI), and its enrichment for genes involved in metabolism, apoptosis, and genome maintenance. However, this remains a hypothesis, and the current evidence is circumstantial rather than definitive. The authors should temper their claims around causality, clearly state that dAD serves as a marker of candidate early events rather than proof of causation and propose experimental validation strategies to substantiate the functional and temporal relevance of the identified dysregulated loci.
The potential for single-cell RNA-seq (scRNA-seq) applications is highly relevant to this work but remains entirely unaddressed. At the single-cell level, allele-specific expression (ASE) can be directly linked to other cellular features such as transcriptional states, CNAs, or differentiation status.

Upon rereading our work, we agree we overplay the causality-aspect a bit much. We have thus tuned this down (especially in the discussion) and included an additional remark that maelstRom, as a computational methodology, remains by itself insufficient to validate such claims and that earliness of dysregulation does not equate causality. The potential of single-cell data in ASE studies also did not go unnoticed, though there are some caveats regarding it applicability to studies as discussed in our manuscript (allelic bursting, the scale and cost of single-cell cohorts). These are now also addressed in the discussion of our revised manuscript.

> R2: When over-dispersion parameter theta is used for over-dispersion estimate in cis-eQTL software overdispersion is quite close to the boundary (same goes for rho). As I understand, here over-dispersion tries to catch more effects and may be generally farther from the boundary, however it is still a question how often the issue of boundary arises. If it happens often, LRT test can't use non-boundary assumtion that easily. Even if authors didn't address this issue in their package, I think the method would be valuable (though it would produce wrong tests for boundary scenarios), but in such case it must be declared as a

current limitation and preferably evaluated. Evaluation could include things such as: how common this issue is and/or argue importance of such cases. Maybe, if the problem is not solvable analytically, some permutation-based method at least for boundary cases would help. Alternatively, authors can argue that such cases are not biologically relevant - whatever their approach is, I think the issue has to be addressed. I see in the text that even for case example in autosomes mean rho is quite low 0.053, which confirms my suspicions that a certain fraction of tests would be even closer to 0. Testing equality of two overdispersions in that scenario would have to be different. In Table 1 authors show select very significant results, however it is a question whether there are some results with, say, rho_control being 0.001 and rho_case being 0.003 or whatever the value has to be to get a significant test result. If there are such results, it would be useful to discuss how much that result can be trusted and if all to be used, maybe such results have to be discarded as not being biologically significant. The point is that this issue has to be discussed and some recommendations for such cases would be useful.

We appreciate this insightful remark; indeed, the here described issue is a common one in many overdispersion-applications, especially those concerned with testing for the occurrence of overdispersion at all (which would, in our case, be rho or theta = 0). Luckily, our (biological) application of the beta-binomial distribution does not struggle with this issue, both theoretically and practically (from some checks we ran, more on that shortly). First off, the boundary case of zero-overdispersion would, biologically speaking, indicate that a given population of RNAseq samples shows only technical variability, i.e. behaves as technical replicates of one identical sample. This is, understandably, not realistic in our studied and heterogeneous population, or most populations for that matter, and we are mostly comparing control- and case-populations which both show sizeable enough overdispersion values (see e.g. Figure 6). That being said, the reviewer understands this and is, specifically, mainly concerned about tests in which one or both overdispersion values get very close to 0, such as 0.001 or 0.003 (both in terms of stability or the LRT and, if it is statistically relevant, biological relevance of the result). In checks we performed on our actual results (e.g. p-value distributions when applying different filter criteria) and experiments on simulated data, we did not observe anomalies in LRT output, perhaps owing to the specific implementation of our likelihood functions (we didn’t explore the theory as to why this is in depth; maybe theta being log10-transformed, thus equaling -3 for values of 0.001 which is still quite far from the boundary cases, helped; theta is also never initialized at zero-values which may, in practice, also help). We also never observed statistically significant dAD results in which both overdispersion values were very low (e.g. the arbitrary example of 0.001 vs. 0.003, as in: both well below mean- or median overdispersion values across our studied population) in real or simulated data. Differences of magnitude at these or similar theta-values do not impact data likelihood enough to return statistically significant tests for realistic gene counts.

That aside, the question regarding which overdispersion effect sizes or differences are biologically relevant, then, is a trickier one; we mainly focused on statistical significance which (as just mentioned, conveniently) never occurred for cases in which both control- and case-overdispersion was very low, though in some places of our manuscript impose a somewhat-arbitrary cutoff of at least a 1.5 rho-ratio in cases versus controls. An issue here, is that our results on one cohort (KIRC) already showed how what consist of a “high” or “low” overdispersion depends on e.g. clonal origin of the studied tissue (compare the X-chromosome to autosomal chromosomes), tumor purities and sampling strategies; and what consists of a “relevant difference in rhos” may depend on the absolute values of rhos being compared. Further studies (in other tissues, cancers, using single-cell transcriptomics) may alleviate such problems, and we address this effect-size related remark in the discussion of our revised manuscript.

We also still acknowledge the theoretical relevance of the boundary issue remark made be the reviewer, and have addressed this in the methodology section where the LRT gets explained (cautioning against its use in testing for the occurrence of overdispersion). Though not discussed in the paper, we are still exploring this issue further, e.g. via beta-binomial implementations which allow for underdispersion in which the “rho=0” case would no longer be a boundary, but this work is ongoing. If this acknowledgement is, in itself, not sufficient to address these concerns, we can still prepare some supplementary scripts and plots (e.g. p-value distributions, simulations experiments) upon request by a reviewer or the journal.

> R2: I'm curious whether authors have seen cases with rho to be lower in cancer compared to control.

How often does it happen and what do they attribute it to. I'm not sure that this should be detailed in the article discussed, but I do wonder whether they saw such cases and decided not to pursue or whether they didn't see such situations at all.

This does happen, though very rarely. If we apply the filters we used to get the 2142 significant autosomal dAD genes in the paper (1E-3 FDR, $\rho_{case} > 1.5\ \rho_{control}$) but in reverse (1E-3 FDR, $1.5\rho_{case} < \rho_{control}$), we have 57 such cases. It is theoretically not unthinkable this occurs: there are known cases of epigenetic regulation which happens very early in life and is clonally maintained (thus potentially giving a large $\rho_{control}$), but can be lost during aging or disease (e.g. loss of parental imprinting). For various reasons though, maelstRom is not particularly suited to study imprinting (e.g. imprinting is often so extreme that all apparent heterozygosity in the data disappears due to the same allele being monoallellically expressed in all cells of any one individual; no heterozygous data means no modelling), but we do not exclude other epigenetic phenomena to follow a similar pattern or for partial (loss-of-)imprinting to give this kind of AD-decreasing layout. Nevertheless, given the central crux of our study (Figure 1) and their relative scarcity, we chose not to pursue such results further. There aren't any obvious positive controls such as the CNA regions showing uniformly increased AD as immediately seen on exploratory plots for such AD-lowering cases, either, so we erred on the side of caution on this one. Having written this out, we do acknowledge this remark may be of interest to other readers too, thus shortly address it in the revised version of our manuscript.

>R2: A small comment regarding "Some ASE studies relied on the beta-binomial distribution, but then simply incorporated AD as a constant nuisance parameter [7, 63], or used it for other purposes (e.g. to combine per-SNP data to the gene level [64])" - I'm sure that 64 also used over-dispersion as a nuisance parameter, the difference was that it was used at a gene level, instead of a SNP level.

This is indeed a more correct description of the situations, we have adapted our text to reflect this remark.

Editorial Manager®

Cookie Preference Center

We use cookies which are necessary to make our site work. We may also use additional cookies to analyze, improve and personalize our content and your digital experience. For more information, see our Cookie Policy.

You may choose not to allow some types of cookies. However, blocking some types may impact your experience of our site and the services we are able to offer. See the different category headings below to find out more or change your settings.

Allow all

Manage Consent Preferences

Strictly Necessary Cookies

Always active
These cookies are necessary for the website to function and cannot be switched off in our systems. They are usually only set in response to actions made by you which amount to a request for services, such as setting your privacy preferences, logging in or filling in forms. You can set your browser to block or alert you about these cookies, but some parts of the site will not then work.

Functional Cookies

These cookies enable the website to provide enhanced functionality and personalisation. They may be set by us or by third party providers whose services we have added to our pages. If you do not allow these cookies then some or all of these services may not function properly.

Performance Cookies

These cookies allow us to count visits and traffic sources so we can measure and improve the performance of our site. They help us to know which pages are the most and least popular and see how visitors move around the site.

Targeting Cookies

These cookies may be set through our site by our advertising partners. They may be used by those companies to build a profile of your interests and show you relevant adverts on other sites. If you do not allow these cookies, you will experience less targeted advertising.

Cookie List

Clear

Apply Cancel
Consent Leg.Interest

Confirm my choices

Powered by onetrust
