# Supplementary material for: Population-level allelic dispersion modeling by maelstRom yields genome-wide maps of allele-specific dysregulation during early carcinogenesis
Source: Gigascience. 2025 Nov 4;14:giaf125. doi: 10.1093/gigascience/giaf125 (PMC12585351; doi:10.1093/gigascience/giaf125)
Supplement: giaf125_Reviewer_1_Report_Original_Submission — Anelia Horvath -- 4/15/2025 [file giaf125_reviewer_1_report_original_submission.pdf]

Reviewer Report

Title: Population-level allelic dispersion modelling by maelstRom yields genome-wide maps of allele-specific dysregulation during early carcinogenesis.

Version: Original Submission **Date:** 4/15/2025

Reviewer name: Anelia Horvath

Reviewer Comments to Author:

The paper shifts the focus from differential gene expression to allelic dispersion (AD), positioning it as a marker of early, mitotically inherited allele-specific alterations. The authors introduce maelstRom, an open-source R/C++ package, capable of modeling AD and dAD using beta-binomial mixture models, requiring no genotyping data. Overal maelstRom offers a genotype-independent, RNA-seq-only framework to uncover early allele-specific regulatory disruptions in cancer. dAD complements DE and may highlight causally relevant events in tumor evolution, particularly those not observable through conventional transcriptomic analyses. The paper is well written.Major concerns:The authors provide a detailed yet ultimately unconvincing rationale for limiting their study to renal clear cell carcinoma (RCC). While RCC is a compelling model due to its early and clonally stable allele-specific copy number alterations (e.g., 3p loss), it is not unique in this respect. Several other cancersâ€"including breast (BRCA), lung adenocarcinoma (LUAD), prostate (PRAD), colon (COAD), and glioblastoma (GBM)â€"exhibit similarly well-characterized early events such as allelic copy number changes, promoter methylation, and epigenetic silencing, many of which are clonal and mitotically maintained. Applying maelstRom to these additional tumor types would not only provide critical validation of the method's generalizability but would also illuminate tissue-specific versus pan-cancer patterns of early allele-specific dysregulation.The authors should clarify the intended application of their population-based dAD method. Is maelstRom meant to be run once per cohort or cancer type to discover candidate early dysregulated loci, or is the goal to eventually support individual-level predictions (e.g., for biomarker development or risk stratification)? If the latter, it is critical to explain how population-derived dispersion parameters could be applied to classify or interpret single samples. At present, the manuscript does not clearly distinguish between discovery-based cohort-level analysis and translational utility at the patient level, and this distinction is essential for assessing the method's broader relevance.Benchmarking: maelstRom is positioned as unique, yet it would be valuable to compare its performance (e.g., runtime, sensitivity, specificity) to existing ASE frameworks (e.g., MBASED, QuASAR, RASQUAL) where applicable.The model appears statistically substantiated, yet diagnostic outputs (e.g., posterior allele bias plots, overdispersion diagnostics, Cook's distance distribution) are not emphasized. These should be built into the output or visualization layer.What exactly is the application of this population-based method? Is it meant to be run once per cohort/cancer type and then used in individual sample risk prediction? The authors need to clarify better this.It would be highly informative to see a genome-wide overview of dAD using circos plots or comparable genome-scale visualizations, either in the main figures or as supplementary material.Minor:The authors infer that dAD identifies putative causal, upstream events based on its ability to capture clonally maintained allele-specific

dysregulation across a population, its co-localization with known early driver events (e.g., 3p loss, XCI), and its enrichment for genes involved in metabolism, apoptosis, and genome maintenance. However, this remains a hypothesis, and the current evidence is circumstantial rather than definitive. The authors should temper their claims around causality, clearly state that dAD serves as a marker of candidate early events rather than proof of causation and propose experimental validation strategies to substantiate the functional and temporal relevance of the identified dysregulated loci.The potential for single-cell RNA-seq (scRNA-seq) applications is highly relevant to this work but remains entirely unaddressed. At the single-cell level, allele-specific expression (ASE) can be directly linked to other cellular features such as transcriptional states, CNAs, or differentiation status.

Level of Interest

Please indicate how interesting you found the manuscript: Choose an item.

Quality of Written English

Please indicate the quality of language in the manuscript: Choose an item.

Declaration of Competing Interests

Please complete a declaration of competing interests, considering the following questions:

- Have you in the past five years received reimbursements, fees, funding, or salary from an organisation that may in any way gain or lose financially from the publication of this manuscript, either now or in the future?
- Do you hold any stocks or shares in an organisation that may in any way gain or lose financially from the publication of this manuscript, either now or in the future?
- Do you hold or are you currently applying for any patents relating to the content of the manuscript?
- Have you received reimbursements, fees, funding, or salary from an organization that holds or has applied for patents relating to the content of the manuscript?
- Do you have any other financial competing interests?
- Do you have any non-financial competing interests in relation to this paper?

If you can answer no to all of the above, write 'I declare that I have no competing interests' below. If your reply is yes to any, please give details below.

I declare that I have no competing interests

I agree to the open peer review policy of the journal. I understand that my name will be included on my report to the authors and, if the manuscript is accepted for publication, my named report including any attachments I upload will be posted on the website along with the authors' responses. I agree for my

report to be made available under an Open Access Creative Commons CC-BY license (http://creativecommons.org/licenses/by/4.0/). I understand that any comments which I do not wish to be included in my named report can be included as confidential comments to the editors, which will not be published.

Choose an item.

To further support our reviewers, we have joined with Publons, where you can gain additional credit to further highlight your hard work (see: https://publons.com/journal/530/gigascience). On publication of this paper, your review will be automatically added to Publons, you can then choose whether or not to claim your Publons credit. I understand this statement.

Yes Choose an item.
