# Supplementary material for: Population-level allelic dispersion modeling by maelstRom yields genome-wide maps of allele-specific dysregulation during early carcinogenesis
Source: Gigascience. 2025 Nov 4;14:giaf125. doi: 10.1093/gigascience/giaf125 (PMC12585351; doi:10.1093/gigascience/giaf125)
Supplement: giaf125_Revision_2_Report_Original_Submission — Vasyl Zhabotynsky -- 5/5/2025 [file giaf125_revision_2_report_original_submission.pdf]

Reviewer Report

Title: Population-level allelic dispersion modelling by maelstRom yields genome-wide maps of allele-specific dysregulation during early carcinogenesis.

Version: Original Submission **Date:** 5/5/2025

Reviewer name: Vasyl Zhabotynsky

Reviewer Comments to Author:

In general I find this article to be well thought out, solid from the method point of view and that it proposes an approach that may produce additional insights to the researchers in the field. There are few minor things I think it would be better to address directly. Overall, I would recommend it to be published.I also like that the authors did not throw away X chromosome (I'm well aware that people tend to do this because it adds pain for modelling, even though the chromosome itself is very important). I think the fact that authors show how method works in autosomes and in X chromosome is both illustrative and commendable.Few comments:When over-dispersion parameter theta is used for over-dispersion estimate in cis-eQTL software overdispersion is quite close to the boundary (same goes for rho). As I understand, here over-dispersion tries to catch more effects and may be generally farther from the boundary, however it is still a question how often the issue of boundary arises. If it happens often, LRT test can't use non-boundary assumtion that easily. Even if authors didn't address this issue in their package, I think the method would be valuable (though it would produce wrong tests for boundary scenarios), but in such case it must be declared as a current limitation and preferably evaluated. Evaluation could include things such as: how common this issue is and/or argue importance of such cases. Maybe, if the problem is not solvable analytically, some permutation-based method at least for boundary cases would help. Alternatively, authors can argue that such cases are not biologically relevant - whatever their approach is, I think the issue has to be addressed. I see in the text that even for case example in autosomes mean rho is quite low 0.053, which confirms my suspicions that a certain fraction of tests would be even closer to 0. Testing equality of two overdispersions in that scenario would have to be different. In Table 1 authors show select very significant results, however it is a question whether there are some results with, say, rho_control being 0.001 and rho_case being 0.003 or whatever the value has to be to get a significant test result. If there are such results, it would be useful to discuss how much that result can be trusted and if all to be used, maybe such results have to be discarded as not being biologically significant. The point is that this issue has to be discussed and some recommendations for such cases would be useful.I'm curious whether authors have seen cases with rho to be lower in cancer compared to control. How often does it happen and what do they attribute it to. I'm not sure that this should be detailed in the article discussed, but I do wonder whether they saw such cases and decided not to pursue or whether they didn't see such situations at all.A small comment regarding "Some ASE studies relied on the beta-binomial distribution, but then simply incorporated AD as a constant nuisance parameter [7, 63], or used it for other purposes (e.g. to combine per-SNP data to the gene level [64])" - I'm sure that 64 also used over-dispersion as a nuisance parameter, the difference was that it was used at a gene level, instead of a SNP level. Essentially, authors wanted to say that listed

methods used over-dispersion as a nuisance parameter while their method suggests that in cancer it can be used to draw biologic interpretation - it is an important contribution to the methods in RNAseq analysis. I'm quite confident that historically in other fields there were examples when it was important to look at change in variation and knowing how different cancer is compared to healthy samples it is reasonable to apply this approach in this field.Also, would not it be a bit more helpful if the list if references goes in alphabetic order?

Level of Interest

Please indicate how interesting you found the manuscript: Choose an item.

Quality of Written English

Please indicate the quality of language in the manuscript: Choose an item.

Declaration of Competing Interests

Please complete a declaration of competing interests, considering the following questions:

- Have you in the past five years received reimbursements, fees, funding, or salary from an organisation that may in any way gain or lose financially from the publication of this manuscript, either now or in the future?
- Do you hold any stocks or shares in an organisation that may in any way gain or lose financially from the publication of this manuscript, either now or in the future?
- Do you hold or are you currently applying for any patents relating to the content of the manuscript?
- Have you received reimbursements, fees, funding, or salary from an organization that holds or has applied for patents relating to the content of the manuscript?
- Do you have any other financial competing interests?
- Do you have any non-financial competing interests in relation to this paper?

If you can answer no to all of the above, write 'I declare that I have no competing interests' below. If your reply is yes to any, please give details below.

I declare that I have no competing interests

I agree to the open peer review policy of the journal. I understand that my name will be included on my report to the authors and, if the manuscript is accepted for publication, my named report including any attachments I upload will be posted on the website along with the authors' responses. I agree for my report to be made available under an Open Access Creative Commons CC-BY license (http://creativecommons.org/licenses/by/4.0/). I understand that any comments which I do not wish to

be included in my named report can be included as confidential comments to the editors, which will not be published.

Choose an item.

To further support our reviewers, we have joined with Publons, where you can gain additional credit to further highlight your hard work (see: https://publons.com/journal/530/gigascience). On publication of this paper, your review will be automatically added to Publons, you can then choose whether or not to claim your Publons credit. I understand this statement.

Yes Choose an item.
